# A Generic Individual-Based Spatially Explicit Model as a Novel Tool for Investigating Insect-Plant Interactions: A Case Study of the Behavioural Ecology of Frugivorous Tephritidae

**DOI:** 10.1371/journal.pone.0151777

**Published:** 2016-03-21

**Authors:** Ming Wang, Bronwen Cribb, Anthony R. Clarke, Jim Hanan

**Affiliations:** 1 Queensland Alliance for Agriculture and Food Innovation (QAAFI), The University of Queensland, Brisbane, QLD, Australia; 2 Centre for Microscopy and Microanalysis, The University of Queensland, Brisbane, QLD, Australia; 3 School of Biological Sciences, The University of Queensland, Brisbane, QLD, Australia; 4 School of Earth, Environmental and Biological Sciences, Queensland University of Technology, Brisbane, QLD, Australia; 5 Plant Biosecurity Cooperative Research Centre, Bruce, ACT, Australia; CNRS, FRANCE

## Abstract

Computational modelling of mechanisms underlying processes in the real world can be of great value in understanding complex biological behaviours. Uptake in general biology and ecology has been rapid. However, it often requires specific data sets that are overly costly in time and resources to collect. The aim of the current study was to test whether a generic behavioural ecology model constructed using published data could give realistic outputs for individual species. An individual-based model was developed using the Pattern-Oriented Modelling (POM) strategy and protocol, based on behavioural rules associated with insect movement choices. Frugivorous Tephritidae (fruit flies) were chosen because of economic significance in global agriculture and the multiple published data sets available for a range of species. The Queensland fruit fly (Qfly), *Bactrocera tryoni*, was identified as a suitable individual species for testing. Plant canopies with modified architecture were used to run predictive simulations. A field study was then conducted to validate our model predictions on how plant architecture affects fruit flies’ behaviours. Characteristics of plant architecture such as different shapes, e.g., closed-canopy and vase-shaped, affected fly movement patterns and time spent on host fruit. The number of visits to host fruit also differed between the edge and centre in closed-canopy plants. Compared to plant architecture, host fruit has less contribution to effects on flies’ movement patterns. The results from this model, combined with our field study and published empirical data suggest that placing fly traps in the upper canopy at the edge should work best. Such a modelling approach allows rapid testing of ideas about organismal interactions with environmental substrates *in silico* rather than *in vivo*, to generate new perspectives. Using published data provides a saving in time and resources. Adjustments for specific questions can be achieved by refinement of parameters based on targeted experiments.

## Introduction

Computational modelling is playing an increasingly significant role in understanding complex biological behaviours. Over a decade ago medical molecular and cell biologists found that computational models, used to simulate hypothesised mechanisms underlying processes in the real world, could be of great value in understanding the systems they study [1]. Such endeavours have also grown in general biology and ecology [2–5].

Individual-based models (IBMs) are a type of computational model that uses simple rules to simulate individual behaviour, the interactions among and between individuals, and the interactions between individuals and their abiotic environment [6]. IBMs simulating individual behaviour have been found to be a valuable tool to analyse the complicated interactions and emergent outcomes observed in behavioural ecology [7–10]. However, IBMs often focus on specific interactions, and require large, specialised biological data sets to derive individual behaviour rules, which can make them difficult to be developed [6].

In cancer research, generic computational models have been constructed using existing data to define foundations for design of patient specific cancer therapy [11, 12]. This idea of using generic models seems not to have been promoted in ecology. In general, IBMs focus on specific questions for which datasets need to be generated [13]. Instead of generating new data before beginning to develop a computational model, biologists or ecologists could be looking at published data and divining commonalities in that data to create generic models; only undertaking additional targeted data-gathering where such models do not match outcomes in specific cases.

To test the feasibility of the above idea, this study focuses on individual-based modelling of the within canopy movement patterns of frugivorous Tephritidae, the ‘true fruit flies’. Because they are pests of economic significance to global agriculture [14], there are multiple data sets available in the published literature on the behaviour of these flies, e.g. for the apple maggot fly, *Rhagoletis pomonella* (Walsh) [15–18], the Chinese citrus fruit fly, *Bactrocera minax* (Enderlein) [19], the European cherry fruit fly, *Rhagoletis cerasi* (Linnaeus) [20], the Queensland fruit fly (Qfly), *Bactrocera tryoni* (Froggatt) [21–25], and others like the Mexican fruit fly, *Anastrepha ludens* (Loew), the melon fruit fly, *Bactrocera cucurbitae* (Coquillett), the oriental fruit fly, *Bactrocera dorsalis* (Hendel), the olive fruit fly, *Bactrocera oleae* (Gmelin), and the Mediterranean fruit fly, *Ceratitis capitata* (Wiedemann) [18].

The success of a generic individual-based spatially explicit model rests on commonality of behavioural rules across the organisms being considered. There is evidence that underlying rules for generating insect movement behaviour patterns in plant canopies may be simple and generic, based largely on plant architecture and some simple insect behavioural rules [26–29]. The same drivers may follow a common pattern for frugivorous fruit flies. Raghu et al. [30] showed that host plant structure has significant effects on the abundance and behaviour of the wild tobacco fruit fly, *Bactrocera cacuminata* (Hering). For the Queensland fruit fly, *B*. *tryoni*, Balagawi et al. [24] and Balagawi et al. [23] argued that traits of plant architecture influence the insect’s interactions with their host plants: most flies were caught in the mid to upper canopy on fruiting plants. The apple maggot fly, *Rhagoletis pomonella* (Walsh) [31], the Mexican fruit fly, *Anastrepha ludens* (Loew) [32], and the Mediterranean fruit fly, *Ceratitis capitata* (Wiedemann) [33], have also been found to have a different abundance at different canopy heights. Although there is evidence showing a significant relationship between host plant architectural characteristics and behaviour of fruit flies, the fundamental scientific questions of how fruit flies optimise their search patterns and limit competition through movement choices and how these movement patterns are affected by plant architecture are still not well understood.

In this paper we hypothesize that spatial patterns of insect abundance in plant canopies emerge from the behaviour of individual insects, so an individual-based model should be able to be used to simulate the studied system [34]. The overall aim of the paper was to develop a generic individual-based spatially explicit model of tephritid within-canopy foraging using published data from many tephritid species, and to determine whether it could then accurately predict the behaviours of a single species (in this case *B*. *tryoni*). Our goal is to determine if IBMs in biology and ecology can only be developed after the generation of unique and targeted data sets, or if at least initial IBMs can be developed using pre-existing data which may or may not be directly related to the particular focus system.

## Materials and Methods

A generic 3D individual-based spatially explicit model was developed, tested and parameterized in terms of the Pattern-Oriented Modelling (POM) strategy [35] and protocol [36]. The generic model was built using published frugivorous tephritid data for multiple species, and aims (1) to predict fruit fly movement and spatial distribution patterns in plant canopies to be compared with the observed patterns in experiments on the behaviour of *B*. *tryoni* in Valencia orange trees, conducted by Dalby-Ball and Meats [22], and (2) to conduct different simulation experiments after being verified. The model description is written in terms of the ODD (Overview, Design concepts, Details) protocol for describing individual and agent-based models [37, 38]. NetLogo [39] has been used to develop the generic 3D individual-based spatially explicit model (for NetLogo 3D 5.1.0 code; see S1 Appendix).

### ODD protocol

#### Overview

**Fig 1:**
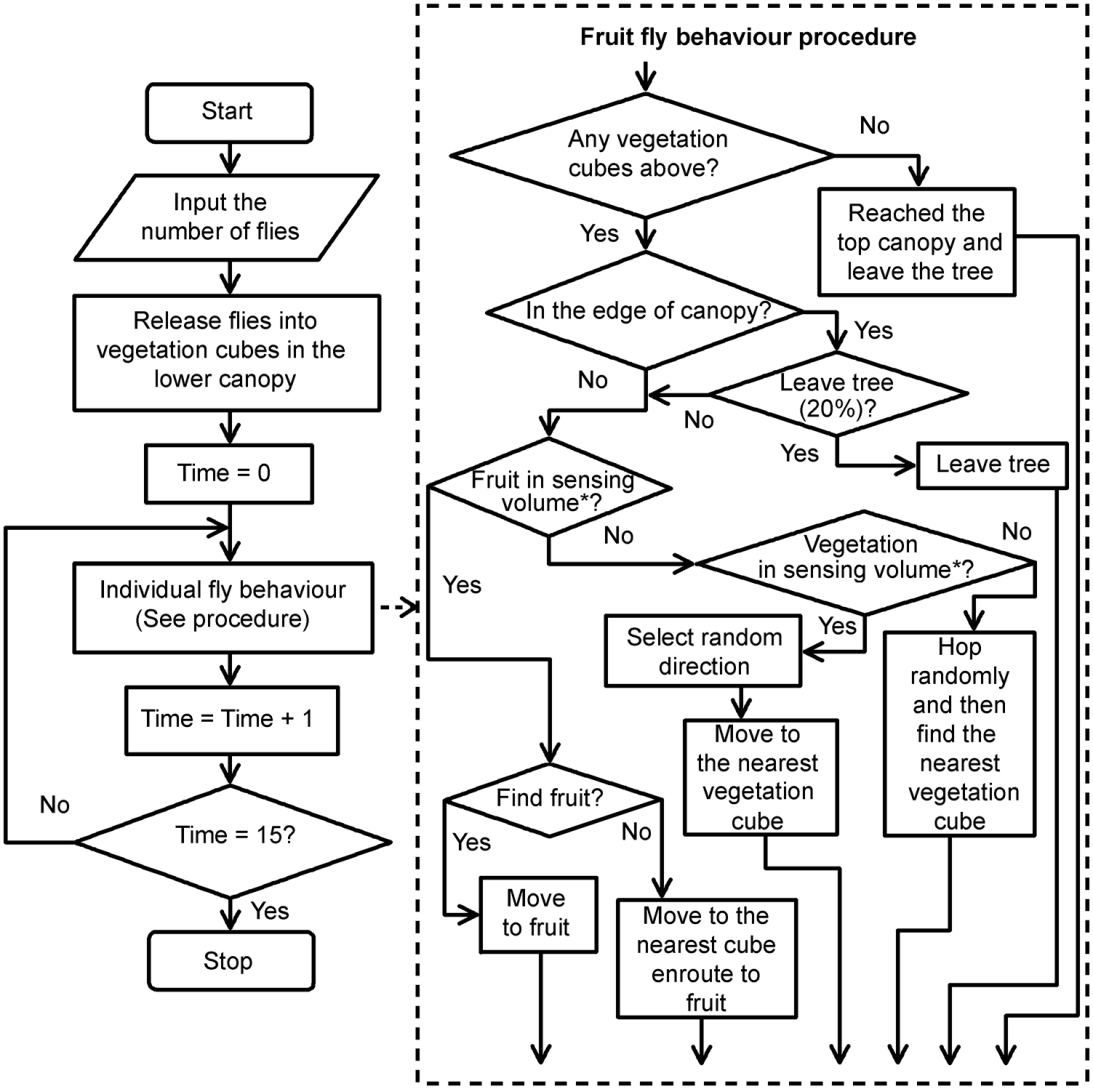
The algorithm flowchart. The algorithm flowchart of generic individual-based spatially explicit fruit fly model for movement within a simulated tree canopy with fruit present. * Definition of sensing volume can be found in the Sensing section of Design Concepts.

1 **Purpose:** The purpose of the model is to simulate movement patterns and spatial distributions of visits (e.g. across canopy regions and across trees) of the frugivorous Tephritidae on foliage and fruits within fruiting plant canopies with different architectures.
2 **Entities, state variables, and scales:** The entities in the model are female fruit flies and the spatial units are cubes composing the fruiting tree and the ground. One unit distance in the model is equal to 4cm in reality. The tree is about 1 m in height and 1.5 m in width. The average leaf area of Valencia orange trees is around 20 cm^2^ [40]. We assume that a green cube represents two leaves with the average leaf area of around 20 cm^2^. A red cube represents host fruit. Individual female flies forage for host fruit in the tree canopy and are characterized by the state variables of identification number, location, and orientation. Foliage and fruit cubes are characterized by the state variables of component type (represented as colour), location and visits counter. The extent of the model worlds are 64 × 64 × 50 cubes. The simulation will stop when the time step reaches 15 (equivalent to 15 minutes in reality).
3 **Process overview and scheduling:** The processes are executed as described in Fig 1. At the beginning of the simulation, a tree is created and female fruit flies are randomly located in green vegetation cubes in the lower third part of tree. In each movement step, if there is any host fruit within the sensing volume, the fruit fly will move in the direction of the fruit landing on the first intervening green cube or on the fruit if there are no cubes between the fruit and itself. If not, the fruit fly will then move to the nearest vegetation cube in a randomly selected direction within the sensing volume. During the simulation the following main processes are executed:
  - *move-flies*: fruit fly undertakes short hops and tends to move upwards by a short distance between vegetation cubes, while foraging for host fruit.
  - *count-visits*: vegetation cubes will show how many times a visit by a fruit fly has occurred.
  - *display-output*: is run when the simulation stops to show the movement patterns as well as spatial distributions of visits on the canopy.

### Design concepts

4 **Design concepts:**
  - Basic principles
    The basic principle of the model is that spatial patterns of insect abundance in plant canopies emerge from the local behaviour of individual insects. Fundamental fruit fly behaviours were derived from the literature for multiple species. Fruit flies tend to move upward by short hops, e.g. the apple maggot fly, *R*. *pomonella* [15], and prefer moving in the inner region of canopy e.g. the Mediterranean fruit fly, *C*. *capitata* and Qfly, *B*. *tryoni* presumably because the intervening foliage can provide flies with some protection from predation [22, 41, 42]. The wild tobacco fruit fly, *B*. *cacuminata* also prefer moving toward denser foliage volumes in the host plant canopy, for the same reason [30], while adjacent foliage increases the probability of finding host fruit for Qfly, *B*. *tryoni* in the tree canopy [25].
    We make the simplifying assumption that local movement to nearest leaves is driving the process. Therefore, the rationale underlying the model is biased random movement, based on environmental inputs including plant component location relative to the fly’s position at any time.
  - Emergence
    The movement patterns and spatial distributions of visits (on foliage and fruit) emerge from mechanistic representation of the behaviours of individual fruit flies, which are not imposed by rules that force the model to produce certain patterns. These patterns are at the population scale.
  - Sensing
    Frugivorous Tephritidae (fruit flies) evaluate the surrounding space for sensory information using principally their eyes and the olfactory hairs and pegs found on the antennae that act as a nose. There is a limit, however, to how far a fly can see both in the forward direction and in an arc around the head (‘field of view’). This also depends on what is being viewed, such as near-by foliage, or the edge of a tree canopy. Each sensory system has a limit to the linear effective range over which it can be active, resulting in a ‘detection radius’ (sensory range). For example, a fly may not be able to see a fruit because of foliage blocking the view, but if the fruit is within a certain distance–the olfactory range–it will be able to smell that a fruit is nearby and move in the direction of the odour. The area within which a fly effectively uses its senses is a volume described by linear distance and arc (degrees from horizontal) that describes a ‘sensing volume’.
    Fruit flies are potentially aware of the vegetation cubes or fruit within a sensory range in their field of view. Lack or low number of vegetation cubes will indicate when they are within the top or edge of the canopy.
  - Interaction
    There are no interactions between fruit fly individuals in this model. Flies can sense fruit and vegetation components and move towards them. The number of visits on vegetation and fruit components is recorded.
  - Stochasticity
    - Fruit flies are randomly positioned in the lower third part of tree using a uniform distribution at the beginning.
    - If flies reach the edge of the canopy, there is a probability (20%) that flies will leave the tree, because the experiments conducted by Dalby-Ball and Meats [22] have shown that some *B*. *tryoni* depart from the fruiting tree before reaching the top of the canopy.
    - To define their orientation, flies will randomly select a target leaf from those within the sensing volume with equal probability.
    - The probability of detecting fruit is defined as a function of distance (see Submodel section).
    - If there is no vegetation cube or fruit within the sensing volume, fruit flies will randomly hop upwards using uniform random numbers to generate hop length and direction within user-defined ranges.
  - Observation
    The number of visits to each vegetation cube and movement trails among vegetation cubes can be displayed numerically, or visually in the model. This provides output for comparisons between runs of the model using different parameter settings for testing, understanding and analysing fly movement patterns. Data are also written to files for further analysis.

### Details

**Fig 2:**
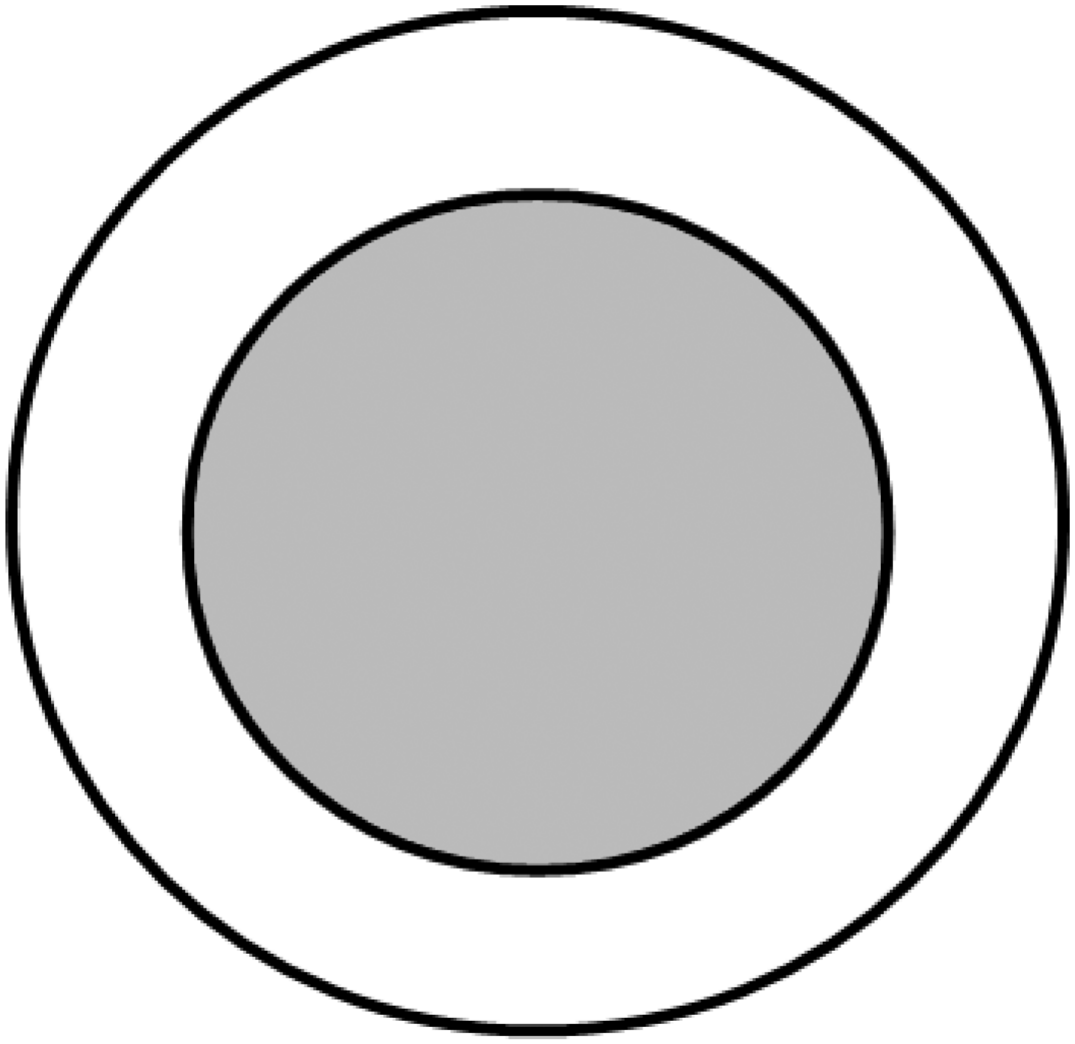
The inner and outer parts of the tree. Concentric circles represent an aerial view of a cross section through the canopy of the simulated tree, divided into two sections, the outer and the inner part of the tree with the same number of vegetation cubes in each of the two sections. The grey-coloured area represents the inner part of the tree.

5 **Initialization:** The extent of the model world is 64 × 64 × 50 cubes. The model world has a tree (tree architecture can be modified for simulation requirements) and a ground made up of cubes (patches in the spatial units of the NetLogo world). A green cube represents two leaves, while a red cube represents host fruit. The tree has a closed-canopy (cylinder volume—height: 1 m and width: 1.5 m) containing 300 green vegetation cubes (equivalent to 600 leaves in reality) and 6 host fruit to be consistent with the experiments conducted by Dalby-Ball and Meats [22]. The foliage density of closed-canopy is 339.53 leaves per cubic meter. The canopy is considered to consist of lower, middle and upper parts, each approximately one-third the height of the canopy and containing 100 green vegetation cubes and 2 host fruit. The canopy is also considered to consist of inner and outer parts (Fig 2), with 150 green vegetation cubes in each. Female flies are randomly placed in green vegetation cubes in the lower third of the tree. The number of visits for all green vegetation cubes and host fruit is set to zero.

**Fig 3:**
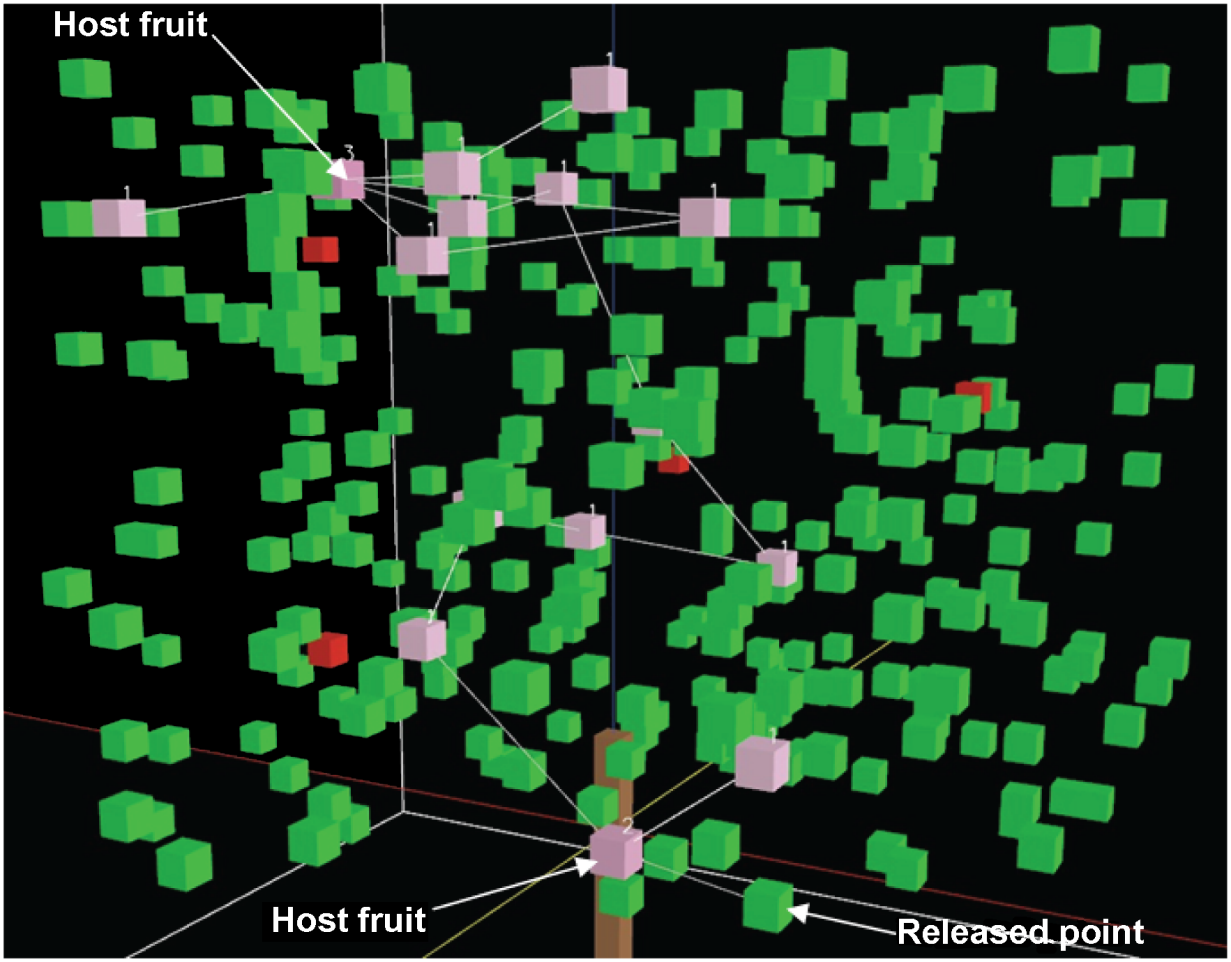
Simulation of individual fly movement through a closed-canopy. Pink cubes show where the fly has already visited with the number of visits, green cubes represent leaves without visits and red cubes represent host fruit without visits. The lines between pink cubs are hop paths.

**Fig 4:**
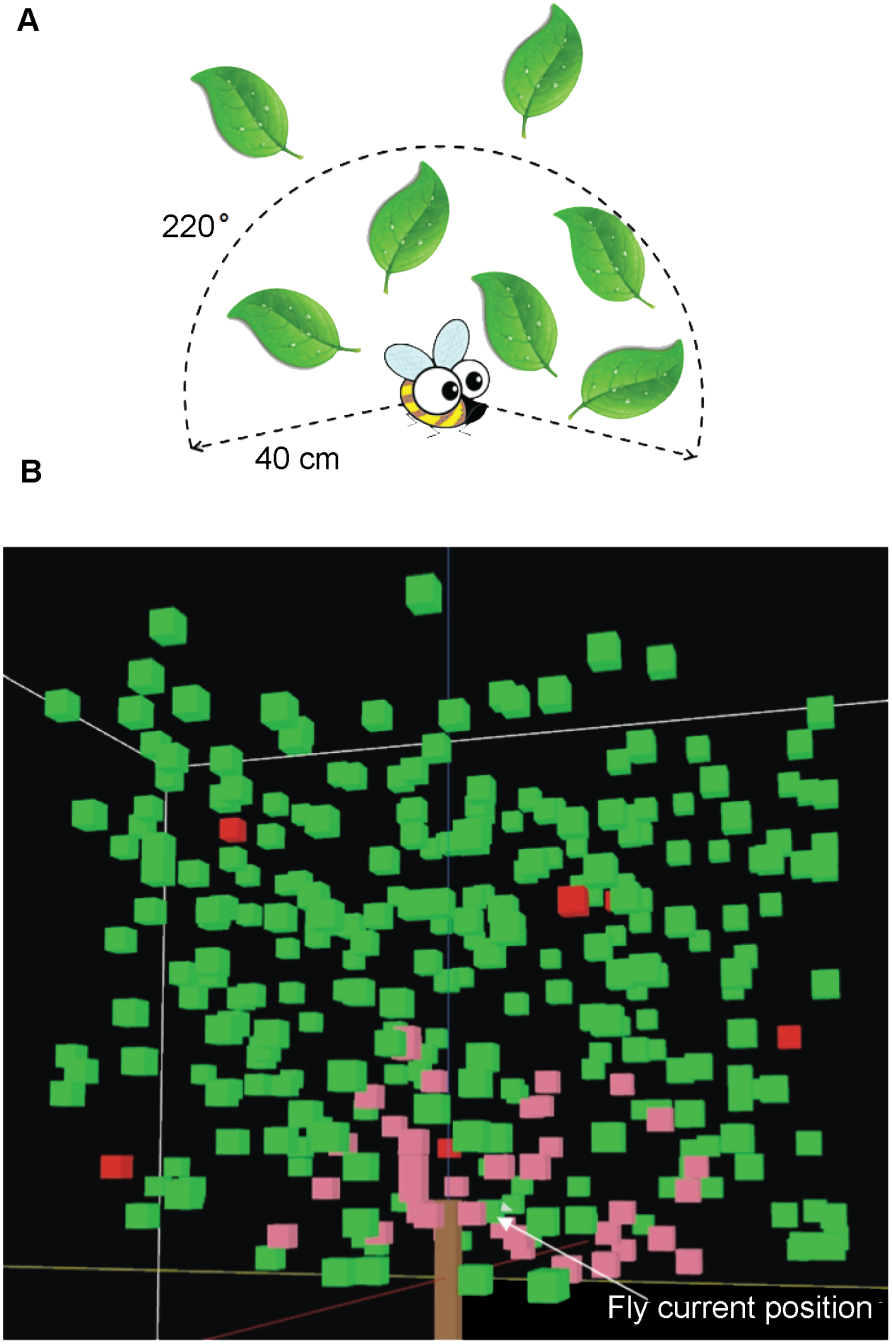
Examples of the sensing volume of an individual fly. A: the 2D representation of sensing volume for an individual fruit fly with 40 cm detection radius and 220° sensing angle. B: an example of the sensing volume of an individual fly in the 3D model. The pink cubes are located within the sensing volume in the closed-canopy with 40 cm and 220 degrees as detection radius and sensing angle respectively. Green cubes represent leaves outside of the sensing volume and red cubes simulate host fruit.

6 **Input data:** No input data.
7 **Submodels:** All model parameters are listed in Table 1.
*move-flies*: The following rules are applied by the individual fruit fly in the simulation (Fig 3):
  - A fly moves by short hops (a limited distance movement between vegetation cubes within the sensing volume)
  - A short hop ends with the fly finding host fruit or the nearest vegetation cube in the previously selected random direction.
  - If flies reach the edge of the canopy, there is a fixed probability of leaving.
  - If not at the top of the canopy, the hopping process is repeated.
  - When the top of the canopy is reached, the fly will leave the tree [22].
A sensing volume is defined by two parameters, the detection radius (sensory range) and sensing angle (field of view). In the current study, we used 40 cm and 220 degrees as detection radius and sensing angle for the sensing volume (Fig 4). The reason for using these values is that:
  - The distance threshold of the sphere of attraction for the apple maggot fly, *Rhagoletis pomonella* (Walsh), is around 80 cm in an apple tree canopy [17]. However, when 80 cm was used in the model, a pattern was produced with too many flies remaining in the tree, which did not match with published experimental outcomes, where according to Dalby-Ball and Meats [22], most *B*. *tryoni* left the tree within 15 minutes. The model identified that the distance threshold of the sphere of attraction is a key parameter in simulating fruit flies’ search for host fruit, therefore additional targeted experiments will need to be conducted for *B*. *tryoni*. We followed the POM strategy [35] and protocol [36], and employed a sensitivity analysis for the detection radius parameter and used three different patterns to reduce the uncertainty in the model structure and parameters [43]. After the sensitivity analysis, the distance threshold of the sphere of attraction was modified to 40 cm, because this distance resulted in a better match to all three observed experimental patterns (S2 Appendix).
  - The flies have a probability of locating fruit that is a function of distance, generated by curve fitting using observed probabilities of visiting the stimulus source in Fig 4(a) from Verdeny‐Vilalta et al. [17]. Flies do not have a 100% chance, as there may be intervening objects. After logistic curve fitting (adjusted R^2^ = 0.879, P < 0.001), a mathematical function was obtained:
    y=a−bln( x+c)
    a= 163.659
    b= 64.852
    c= 1.636
    where *y* is the probability of locating fruit and *x* is the distance between fly and host fruit. For example, if the distance between the fly and host fruit is 10 cm in this tree canopy, the fly will have a 71% chance of finding the host fruit.
  - To model upward and downward movement choices, a 220 degree field of view considering a vertical view direction was arbitrarily chosen in order to be consistent with observations that fruit flies tend to move upward [15, 22], but still allow the possibility of a downward move.
*count-visits*: The number of visits in a vegetation cube will be incremented when a fruit fly arrives in it. Each vegetation cube will show how many times a fly has visited in total.
*display-output*: This process will display gradient-coloured outcomes for the number of visits to each vegetation cube, and the total number of visits in upper, middle and lower canopy, as well as in the inner and outer part of the canopy.

**Table 1:** Overview of parameters in the generic individual-based spatially explicit model for fruit fly movement within a simulated tree canopy with fruit present.

Parameter	Description	Value and Units	References
Pitch			
	Fruit flies face upwards when sensing fruit/foliage	90°	–
	Fruit flies parallel with the ground	0°	–
Field of view			
	For sensing fruit	360°	[17]
	For sensing foliage	220°	[15]
	For sensing the top part of canopy	180° (wide)	–
	For sensing the edge part of canopy	60° (narrow)	–
	For finding the nearest leaf in the direction they are facing	30° (very narrow)	–
Probability			
	The probability that a fly will leave the tree, if it reaches the edge of the canopy	20%	Based on [22]
	The probability of locating fruit—a function of distance: y=a−bln( x+c), where *y* is the probability and *x* is the distance between fly and host fruit	a = 163.659, b = 64.852, c = 1.636	[17] See following explanation in this section
Detection radius			
	The maximum visual and olfactory perceptual distance for a fly to detect foliage and fruit during short hops	40 cm	Based on [17] and sensitivity analysis (S2 Appendix)
Time			
	Time spent on fruit/a leaf by a fly	1 min	Based on [22]
	Searching time spent on the tree by a fly	15 min	Based on [22]

### Model Assessment

The model was assessed using observed patterns in terms of the POM strategy [35] and protocol [36], and using a combination of model evaluation and validation (the ‘evaludation’ approach [44]). The main purpose was to determine whether our model reproduced patterns at multiple scales that have been observed in the field.

#### Data Evaluation

Most parameters of fruit flies in the model have been directly derived from empirical data published in the literature. The parameter of detection radius for fruit flies was calibrated through sensitivity analysis (S2 Appendix). Qualitative and quantitative observed patterns were also used to design the overall model structure.

#### Conceptual Model Evaluation

The conceptual model is represented in Fig 1. The design concepts and simplifying assumptions underlying model design are presented in the ODD protocol section.

#### Implementation Verification

To make sure that the computer code implementing the model works according to its specification in the ODD model description, we have performed a series of tests. Those tests included syntax checking of codes, visual testing through the NetLogo interface, the use of print statements, spot tests with agent monitors, stress tests with extreme parameter values, test procedures, test programs, code reviews, statistical analysis of file output, and independent reimplementation of submodels [45].

#### Model Output Verification

The term ‘verification’ refers to the comparison of model outcomes with observations based on the data the model was tuned for. The model used one fly for each simulation in order to be consistent with the experiments conducted by Dalby-Ball and Meats [22], and has been run 100 times and outcomes used for verification against observed patterns from experiments. This allows us to evaluate the accuracy of specific model processes and outcomes on lower model levels, as it is of significance to the evaluation of IBMs that correct population properties at higher levels can only emerge from a sound base of implemented process and interrelations [46].

Each movement (resulting in a visit) has been set to an equivalent of 1 minute, because the mean time spent by individual wild *B*. *tryoni* observed by Dalby-Ball and Meats [22] was about 1 minute on a leaf or fruit. Therefore, the total number of visits in each region will be the total time spent in that region.

In order for the model to reproduce three patterns observed by Dalby-Ball and Meats [22] at multiple scales, one parameter (detection radius) was inversely determined by sensitivity analysis (S2 Appendix). Using this fine-tuned parameter, our model outcomes match all observed patterns (see Results section).

#### Model Analysis

A sensitivity analysis was performed to explore the behaviour of the model in response to variations in a range of values of the detection radius parameter that was not directly determined from the published literature (S2 Appendix). A robustness analysis [47] was performed to identify conditions under which the modified model no longer reproduces one or more of the patterns, and to improve our understanding of the control mechanisms of the model (S2 Appendix).

#### Model Output Corroboration

The term ‘corroboration’ is defined as the comparison of model predictions with independent patterns that were not used and preferably not even known, when the model was developed, parameterised and verified. After running simulation experiments, our model predictions were compared to independently generated findings of other studies on *B*. *tyroni*, with which they are consistent (see Results section). Two patterns were identified from the published literature and one pattern is derived from our field study (S3 Appendix). Such secondary or independent predictions can be strong indicators that the model is structurally realistic. By comparing model outputs to multiple patterns observed in the field or experiments we can increase our confidence that the model performed well [43].

### Simulation Experiments for Model Corroboration

#### Simulation experiment 1: different tree architecture

**Fig 5:**
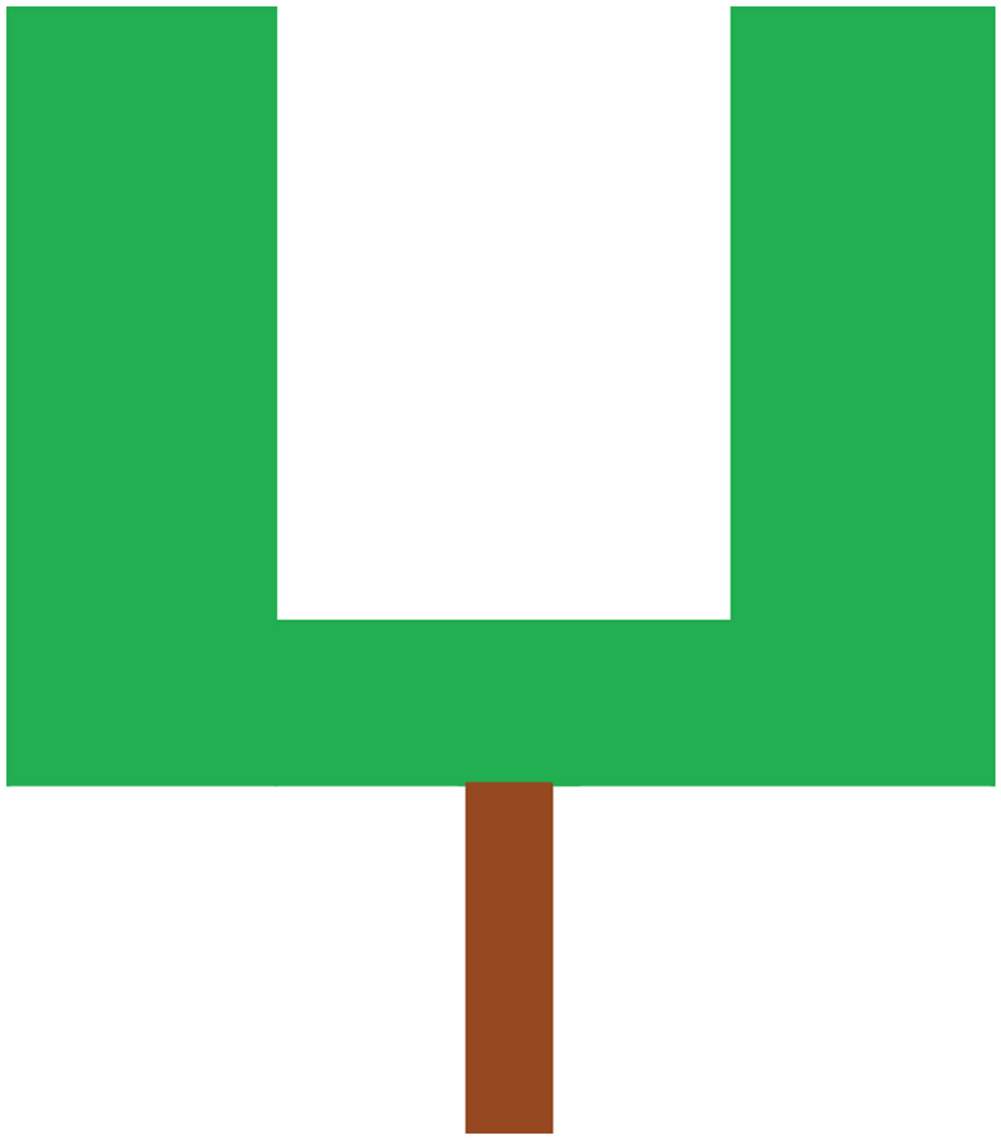
The vase-shaped tree. A simple cross sectional view of the vase-shaped canopy, where the foliage cubes are represented by the green coloured areas and the brown colour area represents the trunk.

To explore the capacity of the generic model to generate new perspectives on *B*. *tryoni* behaviour in different types of tree architecture, a vase-shaped tree has been created for simulation. This is similar to the closed-canopy tree, but vegetation cubes have been removed from the central part of canopy (Fig 5) as is done in some Australian orchards [48, 49]. The vase-shaped canopy is considered to consist of lower, middle and upper parts, each approximately one-third the height of the canopy and containing 75 green vegetation cubes and 2 host fruit. This simulation experiment will be used to examine whether such modified architecture can change movement and abundance patterns of *B*. *tryoni*.

#### Simulation experiment 2: different fruit position

The host fruit in the closed-canopy was positioned in the edge region of the lower to upper canopy initially. To investigate whether the position of host fruit in the canopy can be an important factor affecting movement choices and host-finding of *B*. *tryoni*, a simulation was run with all host fruit placed in the central parts of the lower to upper canopy.

#### Simulation experiment 3: different entry point of *B*. *tryoni*

The entry point of *B*. *tryoni* to a tree canopy can be crucial if they are in a field situation such as an orchard. In the experiments [22], the *B*. *tryoni* were released in the lower canopy as the starting point and therefore the fruit in that region might be more likely to be found. For comparison, two scenarios have been simulated for *B*. *tryoni*: 1) release into the lower canopy and 2) release into the upper canopy. Both will use the closed-canopy tree, in order to find out whether entry position affects subsequent behaviour and visit patterns.

### Data Analysis

Statistical analysis of visits/time in the different geometrical zones, e.g. the upper, middle, lower, inner, and outer canopy, was undertaken. Geometrical zones were included in the analysis because distribution of flies was known to differ on the basis of height within canopy, as obtained from published real-world studies [24, 30–33]. For each simulation experiment, the model was run with 30 flies, and replicated 50 times. Treatments for the three simulation experiments were: tree shape (vase-shaped, versus closed-canopy as the control); position of fruit (centre positioning of fruit, versus edge as the control); and entry point (upper, versus lower as the control). Each run was set to stop when the time step reached 15 (equivalent to 15 minutes in reality). Model outputs have been checked for normality using the Shapiro-Wilk normality test. Depending on the result, either the Kruskal-Wallis test or the one-way ANOVA followed by the Tukey test was used to test for significant differences among regions. A paired t-test or the Wilcoxon Signed-Rank test was applied to test for significant differences between two regions. Treatments were compared using the independent samples t-test or the Mann-Whitney-Wilcoxon test. If there were no visits in any region of canopy, the one-way tables Chi-square test and two-way tables Chi-square test were used to assess differences for the proportion of visits per canopy region within the canopy and among treatments respectively.

## Results

### Model output verification

Three patterns observed by Dalby-Ball and Meats [22] were used for verification (Table 2).

**Table 2:** The three observed patterns used for model output verification.

Patterns	Description
Pattern1	The most visits occur in the inner part of the tree
Pattern2	Most flies (over 80%) leave the tree within 15 minutes
Pattern3	The mean number of visits per fly on tree foliage is 8.9 (SD = 4.802) found in experiments

The model outcomes show:

- Pattern1: the most visits occur in the inner part of the tree: mean = 5.12 (SD = 3.627) for inner part and 3.72 (SD = 2.519) for outer part (t = 3.043, df = 99, P = 0.003).
- Pattern2: most flies (81%) leave the tree within 15 minutes.
- Pattern3: the mean number of visits per fly on tree foliage is 8.84 (SD = 4.223), which is similar to 8.9 (SD = 4.802) found in experiments (t = 0.064, df = 125, P = 0.949).

These model outcomes are consistent with observations in the experiments.

### Simulation experiment 1: different tree architecture

**Fig 6:**
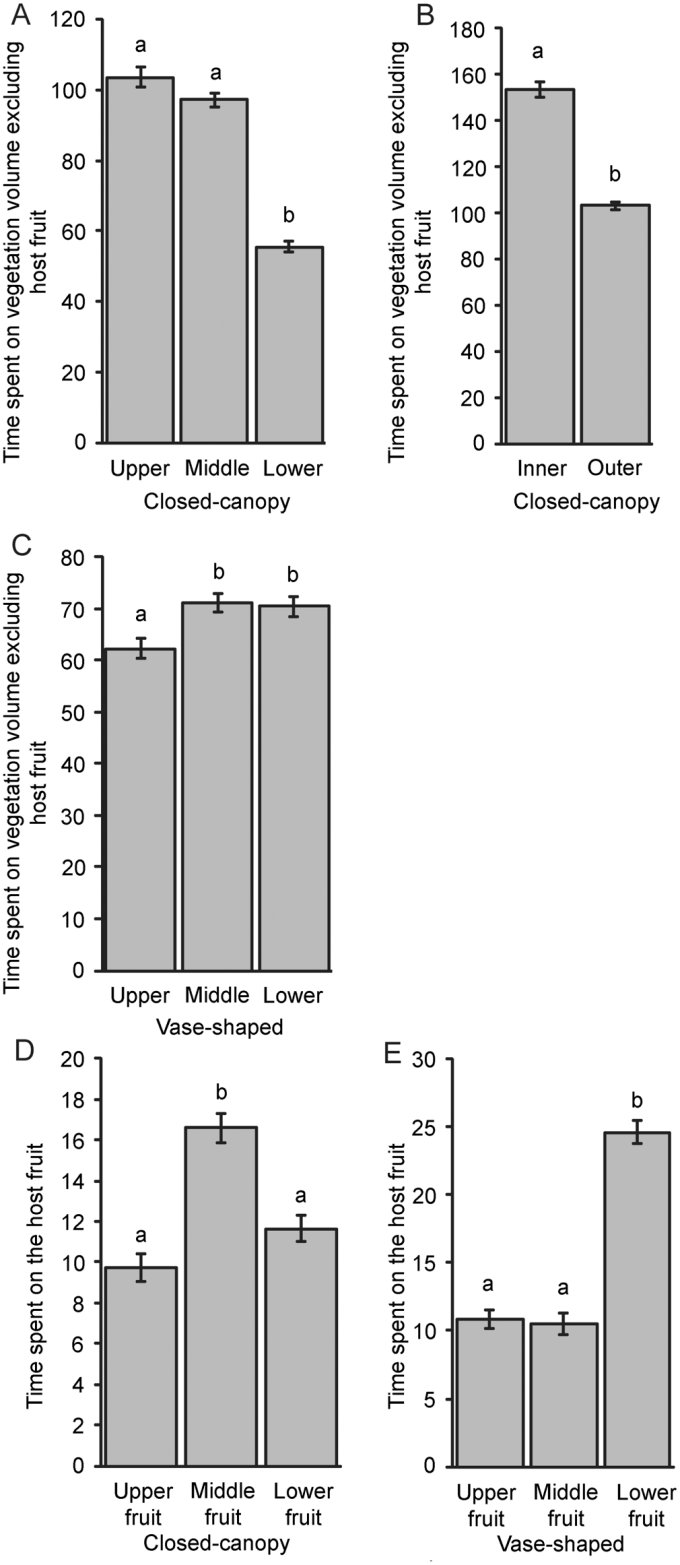
The results of closed-canopy tree and vase-shaped tree treatments. Mean time (min) spent by 30 *Bactrocera tryoni* on the vegetation volume and host fruit in each part of two types of tree canopy. Columns surmounted with the same letter are not significantly different at P = 0.05 (n = 50 simulations). A: upper vs. middle vs. lower (Mean = 103.70|97.24|55.58, F = 138.42, df = 2, P < 0.001). B: inner vs. outer (Mean = 153.48|103.04, t = 13.67, df = 49, P < 0.001). C: upper vs. middle vs. lower (Mean = 62.32|71.1|70.44, F = 6.67, df = 2, P = 0.002). D: upper vs. middle vs. lower (Mean = 9.74|16.6|11.64, F = 26.53, df = 2, P < 0.001). E: upper vs. middle vs. lower (Mean = 10.84|10.5|24.62, F = 111.37, df = 2, P < 0.001).

In the closed-canopy, the time spent on green vegetation cubes in upper or middle regions was almost twice the time spent on green vegetation cubes in the lower region. The time spent on green vegetation cubes in the inner region was 1.5 times as long as the time spent on green vegetation cubes in the outer area. These data were found to differ significantly from one another (Fig 6).

For the time spent on host fruit in the closed-canopy, the middle region showed the longest time among three regions. It was found to differ significantly from the time spent on host fruit in upper or lower regions in the closed-canopy (Fig 6).

In the vase-shaped tree architecture, more time was spent on green vegetation cubes in the middle or lower regions than in the upper region. The time spent on green vegetation cubes for upper vs. middle or upper vs. lower comparisons were found to differ significantly from one another (Fig 6). There was no significant difference between data for middle and lower regions. There was no ‘inner part’ in the vase-shaped architectural simulations (Fig 5).

Flies spent twice as long on host fruit in the lower region of the vase-shaped tree, compared with upper or middle regions. These data were found to differ significantly from one another (Fig 6).

Comparison of time spent in different locations among the two trees showed significant differences (Table 3). Flies spent more time in the upper region of the closed-canopy than in the vase-shaped tree: the time spent on green vegetation cubes in the upper part of the closed-canopy was almost twice that of in the upper part of the vase-shaped tree. Similarly, the time spent on green vegetation cubes in the middle part of the closed-canopy was longer than in the middle part of vase-shaped tree, whereas in contrast, the time spent on green vegetation cubes in the lower part of the closed-canopy was shorter than in the lower part of vase-shaped tree.

**Table 3:** The comparison of mean time (min) spent by 30 *Bactrocera tryoni* on the vegetation volume and host fruit in each part of the two types of tree canopy.

	Closed-canopy	Vase-shaped			
	Mean time		t	df	P
**Upper**	103.7	62.32	11.975	84.412	**< 0.001**
**Middle**	97.24	71.1	9.761	96.58	**< 0.001**
**Lower**	55.58	70.44	-5.864	92.094	**< 0.001**
**Fruit in upper**	9.74	10.84	-1.133	97.868	0.26
**Fruit in middle**	16.6	10.5	5.804	97.9	**< 0.001**
**Fruit in lower**	11.64	24.62	-12.271	90.198	**< 0.001**
**Total fruit**	37.98	45.96	-4.753	96.75	**< 0.001**

Note: the data sets for time spent on green vegetation cubes and host fruit in each part of two types of tree architecture were normally distributed (Shapiro-Wilk normality test). An independent samples t-test was used to compare time spent on the green vegetation cubes and host fruit in the same parts of the canopy but between different architectures (e.g. the upper part of closed-canopy vs. the upper part of the vase-shaped tree).

For the time spent on host fruit, the time spent in the middle in the closed-canopy was longer than the time spent in the middle in the vase-shaped tree, whereas the time spent in the lower region in the vase-shaped tree was twice as long as the time spent in the lower region in the closed-canopy. The time spent in total on fruit in the closed-canopy was shorter than in the vase-shaped architecture. These data were found to differ significantly from one another (Table 3).

### Simulation experiment 2: different fruit position

**Fig 7:**
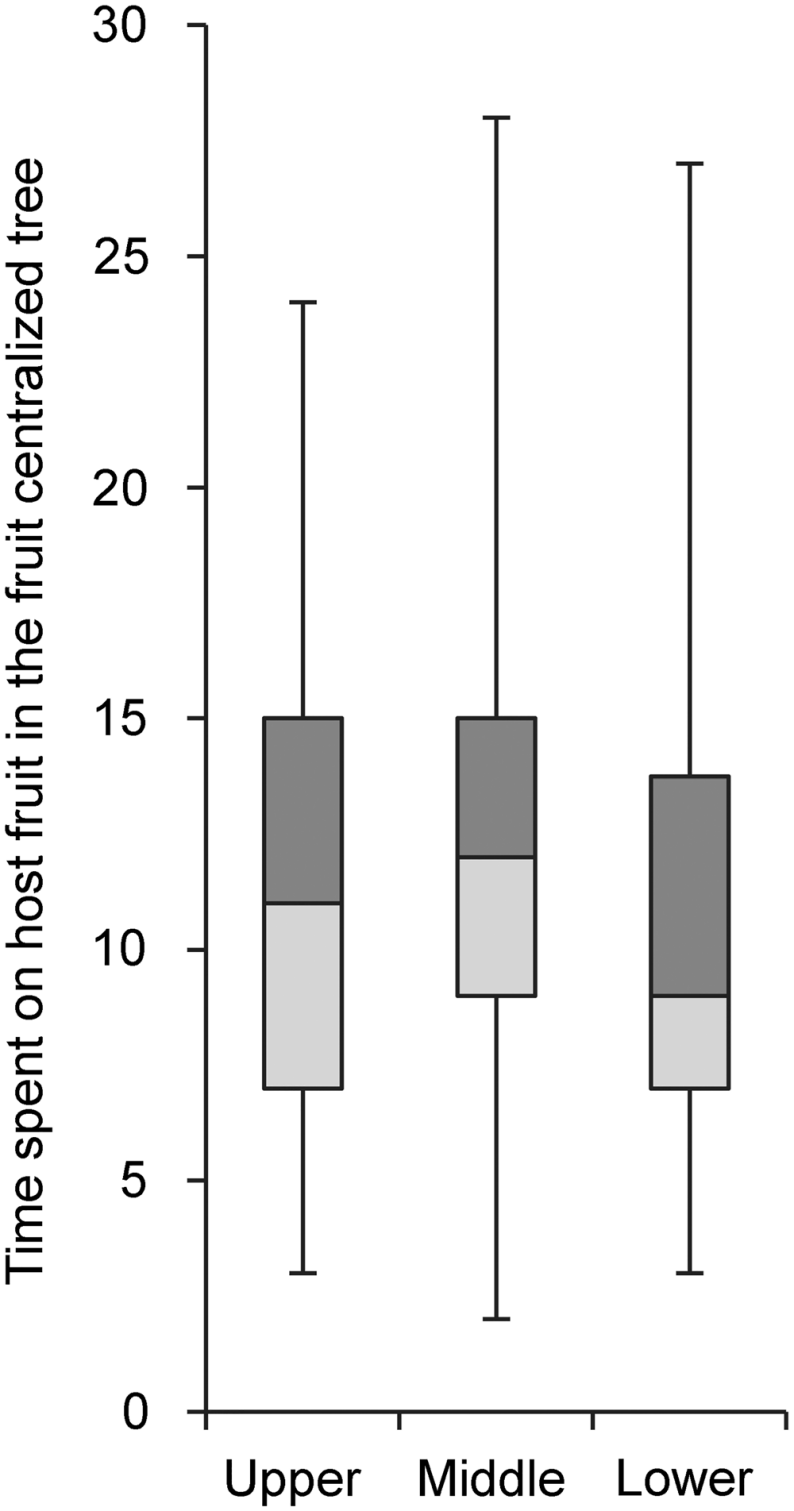
The results of fruit-centralized tree treatment. Median time (min) spent by 30 *Bactrocera tryoni* on host fruit in each part of the fruit-centralized tree (Kruskal-Wallis test: Median = 11|12|9, H = 5.43, df = 2, P = 0.066, n = 50 simulations).

By using two tree models each with different fruit distributions we were able to compare time spent on fruit in a centralised position versus fruit arranged at the edge. Firstly, the time spent in upper, middle and lower regions on fruit were analysed. Data for fruit distributed in the edge region were presented in simulation experiment 1. Data for fruit distributed centrally is shown in Fig 7. More time was spent on fruit when it was distributed only in the edge region than on fruit when it was clustered only centrally. These data were found to differ significantly from one another (Table 4).

**Table 4:** The comparison of mean/median time (min) spent by 30 *Bactrocera tryoni* on host fruit in each part of two treatments.

	Closed-canopy	Fruit centralized tree	Closed-canopy	Fruit centralized tree				
	Mean time		Median time		t	W	df	P
**Fruit in upper**	9.74	11.52	-	-	-1.692	-	96.695	0.094
**Fruit in middle**	16.6	12.46	-	-	3.925	-	97.851	**< 0.001**
**Fruit in lower**	-	-	12	9	-	1559	-	**0.033**
**Total fruit**	37.98	34.14	-	-	1.999	-	88.749	**0.049**

Note: an independent samples t-test was used to compare time spent on host fruit in the same parts of the canopy but between two treatments (e.g. the upper part of closed-canopy vs. the upper part of the fruit centralized tree), while the Mann-Whitney-Wilcoxon Test that is the non-parametric equivalent of the independent samples (or two-sample) t-test, was used when the data sets are not normally distributed.

### Simulation experiment 3: different entry point of *B*. *tryoni*

**Fig 8:**
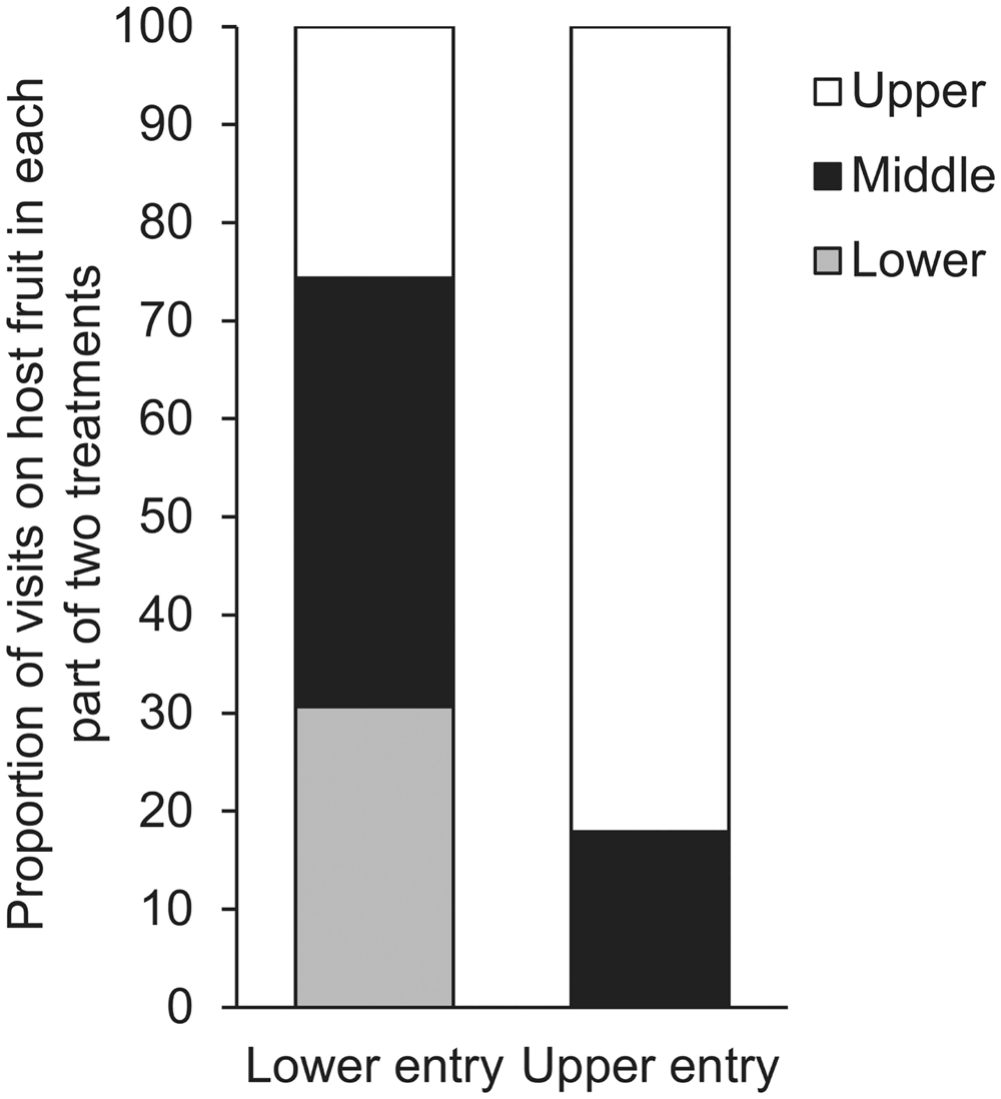
The results of simulation experiment 3: different entry point of *B*. *tryoni*. Visits on host fruit in a divided plant canopy for different entry points by *B*. *tryoni*: data displayed as proportion. A one-way tables Chi-square test and a two-way tables Chi-square test were used to look at the differences in visits per canopy region within the canopy and among treatments respectively: X^2^ = 99.093, df = 2, P < 0.001, n = 50 simulations for the lower entry point; X^2^ = 1010.19, df = 2, P < 0.001, n = 50 simulations for the upper entry point; the two treatments differed significantly: X^2^ = 836.998, df = 2, P < 0.001, n = 50 simulations.

The use of two different entry points for *B*. *tryoni* into the tree canopy (closed-canopy type) allowed an investigation of the importance of this factor on movement and visitation within the canopy. In the tree with the new entry point (upper region entry rather than lower), the visits (742) on host fruit in the upper region was four times greater than in the middle region (162). There were no visits on host fruit in the lower region. The visits on host fruit among three regions in the tree with the new higher entry point or with lower entry point, were significantly different (Fig 8).

For comparison of visits on host fruit among the two treatments, the visits (742) in the upper part of the tree with the higher entry point was 1.5 times as many as the visits (487) in the upper part of the closed-canopy. Not surprisingly, less visits were found on fruit in middle or lower parts in the tree with the new entry point. The visits (904) on total fruit in tree with the new entry point were half that (1899) of the initial lower entry point closed-canopy tree. The visits among three regions on both treatments were significantly different (Fig 8).

### Model output corroboration

Our model predictions were compared to independently generated findings of other studies, after running simulation experiments. Three independent patterns (Table 5) have been used for the model output corroboration.

**Table 5:** The overview of three independent patterns used for the model output corroboration.

Patterns	Description	Source
Pattern4	Fewer *B*. *tryoni* are found in the lower canopy compared with the middle and upper canopy in the closed-canopy tree	[24] (pp. 284)
Pattern5	Different plant architectures (denser, closed canopy vs. open, branched canopy) affect *B*. *tryoni* resting behaviours on foliage and host fruit	[23] (pp. 515) and our field study (S3 Appendix)
Pattern6	*B*. *tryoni* spend more time on host fruit (in total) in vase-shaped canopies	Our field study (S3 Appendix)

The model predictions show:

- Pattern4: fewer *B*. *tryoni* are found in the lower canopy compared with the middle and upper canopy in the closed-canopy tree (Fig 6).
- Pattern5: significant differences for movement patterns of *B*. *tryoni* among different types of tree architecture (closed-canopy vs. vase-shaped) (Table 3).
- Pattern6: *B*. *tryoni* spend more time on host fruit (in total) in vase-shaped canopies (Table 3).

These model predictions are consistent with the three independent patterns.

## Discussion

The generic individual-based spatially explicit model, built here from published data on frugivorous tephritids and operating on a simple set of behavioural rules, predicted fruit fly movement and spatial distribution patterns consistent with published literature. Such patterns, secondary or independent predictions, discussed below can be strong indicators that the model is structurally realistic [43]. Our generic model worked for a specific tephritid species against which it was tested, namely *B*. *tryoni*. There is general agreement that the abundance of frugivorous fruit flies at different canopy heights is varied, and the increasing height is generally associated with greater abundance of frugivorous fruit flies [24]. This argument can be supported by data from the wild tobacco fruit fly, *Bactrocera cacuminata* (Hering) [30], the apple maggot fly, *Rhagoletis pomonella* (Walsh) [31], the Mexican fruit fly, *Anastrepha ludens* (Loew) [32], and the Mediterranean fruit fly, *Ceratitis capitata* (Wiedemann) [33]. In particular, the model predictions match with the findings by Balagawi et al. [24] in terms of the patterns of abundance of *B*. *tryoni* in different heights of canopy, in which fewer *B*. *tryoni* are found in the lower canopy compared with the middle and upper canopy. Furthermore, our model predicts that *B*. *tryoni* spend more time in the inner region of canopy. There is evidence that fruit flies prefer moving in the inner region of canopy e.g. the Mediterranean fruit fly, *C*. *capitata* and Qfly, *B*. *tryoni* presumably because the intervening foliage can provide flies with some protection from predation [22, 41, 42]. The wild tobacco fruit fly, *B*. *cacuminata* also prefer moving toward denser foliage volumes in the host plant canopy, for the same reason [30].

The model also generated specific predictions of behaviour. Orchardists modify tree architecture for a number of reasons such as to increase fruit-yield and reduce occurrence of some pests and diseases [48, 50] but impact on tephritid movement and host-finding success does not appear to have been investigated specifically. To investigate differences in movement patterns of *B*. *tryoni*, predictive simulations were run with plant canopies whose architecture had been modified. The output suggests significant differences for movement patterns of *B*. *tryoni* among different types of tree architecture. For example, *B*. *tryoni* were predicted to spend more time on host fruit (in total) in vase-shaped canopies. Changes in canopy architecture also result in *B*. *tryoni* leaving the tree either earlier or later and therefore affect likelihood of fruit-finding. In support of these predictions, Balagawi et al. [23] found that *B*. *tryoni* prefer a more open and branched canopy over a denser and closed canopy. The vase-shaped canopy may be presenting architecture more in accordance with such an open and branched canopy. In addition, our field study demonstrated that host fruit (in total) were more often visited by *B*. *tryoni* in the vase-shaped canopy, in contrast to in the closed-canopy. As a result, *B*. *tryoni* spent more time on host fruit (in total) in the vase-shaped canopy (S3 Appendix).

Raghu et al. [30] suggested that host plant architecture, e.g. density of foliage, and microclimate, e.g. temperature and light intensity, can significantly affect the abundance and behaviour of the wild tobacco fruit fly, *Bactrocera cacuminata*. An increase in numbers of wild tobacco fruit flies at the host plant is associated with increasing temperature and light intensity, and more wild tobacco fruit flies were found in host plants with dense foliage. The data output from the model can be understood from the perspective that a vase-shaped canopy could get more sunlight due to its shape, and fruit flies could therefore spend more time in the vase-shaped tree and this would increase the chance of finding host fruit for flies. So the generic model has generated outcomes that relate to microclimate from simple behavioural movement rules. A more complex model constructed from data integrating multiple environmental factors was not found to be necessary. This outcome supports our hypothesis that initial IBMs can be built successfully from existing data focussed on fundamental behavioural rules without the need for generating dedicated and complex data sets that can take many years to achieve.

As another example, the model identified that the position of host fruit in the canopy and the entry point of *B*. *tryoni* to a tree canopy may be crucial in terms of fruit finding and number of visits, which would translate to infestation of a crop. The prediction is that *B*. *tryoni* will spend more time on host fruit in the edge region of a canopy, as well as on host fruit in the upper canopy. An application of this model output would be to place traps in fruiting trees in the upper canopy at the edge in closed-canopy trees, and mid to upper parts in vase-shaped architecture in order to maximise fly-catch. This prediction is supported by a study that looked at positioning of protein bait sprays for *B*. *tryoni* management to maximise efficacy and came to the conclusion that they should be applied as high in the canopy as is mechanically possible [24].

Although unique predictions require validation in the real world, the strength of the modelling approach is that trust-worthy predictions can be achieved in a timely fashion from current data. Additionally, such pattern-oriented models can produce comparative predictions that can be tested in the field [35, 36]. As a result, time and resources can be better focussed on experimentation that shows the greatest likelihood of success. For instance, we conducted our field study based on our model predictions, and the findings of our field study are consistent with model predictions (S3 Appendix).

Once a generic model is constructed, however, there may be cause to adjust it further to study particular interactions, and for particular species. In our case, the detection radius of the sphere of attraction is an example of this. Data for how close a fly needs to be to a host-fruit to respond to it is only available for *Rhagoletis pomonella* (Walsh), where it appears to be associated with the density of the tree canopy [17]. The generic model has identified these data as important for future research efforts to better adjust the model to individual species for exploration of foliage density effects. Studies have suggested that animals tend to optimize their foraging activities (e.g. strategies of movement) [51, 52]. Our model also suggested that detection radius for fruit flies in trees with the same foliage density may vary, largely based on relative tree size. In our study, the closed-canopy tree is about 1.5 m diameter by 1 m height and the use of 40 cm (medium short distance compared to the tree height) for fruit flies allows them to find more fruit in the tree (S2 Appendix). However, Verdeny‐Vilalta et al. [17] found that detection radius for *Rhagoletis pomonella* (Walsh) is approximately 80 cm in a tree with about 2.8 m diameter by 3 m height. Thus, the distance for short hops of fruit flies during searching for host fruit in trees with same foliage density but different height, may vary, largely based on the relative host plant size. Future research will need to be conducted to look at this.

Other adjustments are also possible. In the current model, a volume is used to represent vegetation. A finer resolution was not needed because the analysis focused on outputs involving abundance and spatial distribution within architectural canopies. To involve movement patterns at individual leaf or branch level, more detailed modelling of plant architecture can be achieved using the Lindenmayer system (L-system) formalism [53, 54], which can provide an explicit and detailed model of the 3D structure [55–57].

In summary, this study has demonstrated that it is feasible to build an individual-based model from generic published data from multiple species that utilise similar behavioural rules, to explore behavioural ecology of an individual species. Species that hold economic importance generally have multiple published data sets to draw from, in comparison to those that do not. Given this distinction, the generic individual-based spatially explicit model is best suited to driving innovation in ecological studies that have direct application to management issues. For other species, targeted data generation would be necessary, informed by the data sets that have proven most useful for the generation of the current model. Such a model allows rapid testing of ideas about organismal interactions with environmental substrates *in silico* rather than *in vivo*, to generate new perspectives. Since published data are used, there is a saving in time and resources. Adjustments for specific questions can be achieved by tuning parameters based on targeted experimentally obtained data or utilisation of different modelling platforms such as the L-system to simulate at a more detailed level.

## Supporting Information

S1 Appendix NetLogo 3D 5.1.0 Code. (DOCX)

S2 Appendix Model Analysis. (DOCX)

S3 Appendix Field Study. (DOCX)
