# Supplementary material for: A Generic Individual-Based Spatially Explicit Model as a Novel Tool for Investigating Insect-Plant Interactions: A Case Study of the Behavioural Ecology of Frugivorous Tephritidae
Source: PLoS One. 2016 Mar 21;11(3):e0151777. doi: 10.1371/journal.pone.0151777 (PMC4801379; doi:10.1371/journal.pone.0151777)
Supplement: S1 Appendix — (DOCX) [file pone.0151777.s001.docx]

**S1 Appendix: NetLogo 3D 5.1.0 Code**

*breed [flies fly] ; Fruit flies are breeds of turtle.*

*turtles-own [step step-length] ;Fruit flies own some variables*

*flies-own [count-down]*

*patches-own [counter] ;Foliage/fruit variable*

*globals [time fruit-chosen-flag detection-radius lower-start lower-end mid-start mid-end upper-start upper-end] ;global variables*

*to setup ;set up model world*

*clear-all*

*orbit-down 90*

*draw-axes*

*setup-trees*

*setup-flies*

*ask patches with [ pzcor <= -34 ]*

*[ set pcolor random-float 2 + 35 ]*

*reset-ticks*

*end*

*to draw-axes ;Create X, Y and Z axes*

*crt 1 [ set shape "line"*

*set heading 90*

*set color red*

*set size world-width*

*stamp*

*die ]*

*crt 1 [ set shape "line"*

*set color yellow*

*set heading 0*

*set size world-height*

*stamp*

*die ]*

*crt 1 [ set shape "line"*

*set pitch 90*

*set color blue*

*set size world-depth*

*stamp*

*die ]*

*ask patch max-pxcor 0 0 [ set plabel "max x-axis" ]*

*ask patch min-pxcor 0 0 [ set plabel "min x-axis" ]*

*ask patch 0 max-pycor 0 [ set plabel "max y-axis" ]*

*ask patch 0 min-pycor 0 [ set plabel "min y-axis" ]*

*ask patch 0 0 max-pzcor [ set plabel "z-axis" ]*

*ask patch 0 0 min-pzcor [ set plabel "z-axis" ]*

*end*

*to setup-flies ;Create fruit flies*

*create-flies number-fly [*

*set color white*

*random-xyz-cor ; Release flies in the lower part of canopy*

*set size 2*

*set count-down 1 ; visiting time on fruit*

*set step-length 0*

*]*

*end*

*to setup-trees ; Create trees containing lower, middle and upper canopies*

*set lower-start 1*

*set lower-end 8*

*set mid-start 9*

*set mid-end 17*

*set upper-start 18*

*set upper-end 25*

*with-local-randomness [*

*random-seed 126*

*ask patches with [pxcor = 0 and pycor = 0 and pzcor <= 3][ set pcolor 35 ]*

*; *****************************Closed-canopy********************************

*ask n-of 100 patches with [ distancexy 0 0 < ( world-width * 0.75 / 2 - 6) and pzcor >= lower-start and pzcor <= lower-end][ set pcolor lime ]*

*ask n-of 100 patches with [ distancexy 0 0 < ( world-width * 0.75 / 2 - 6) and pzcor >= mid-start and pzcor <= mid-end][ set pcolor lime ]*

*ask n-of 100 patches with [ distancexy 0 0 < ( world-width * 0.75 / 2 - 6) and pzcor >= upper-start and pzcor <= upper-end][ set pcolor lime ]*

*; ******************************* Vase-shaped********************************

*;ask n-of 75 patches with [ distancexy 0 0 < ( world-width * 0.75 / 2 - 6) and distancexy 0 0 > ( world-width * 0.75 / 2 - 15 ) and pzcor >= upper-start and pzcor <= upper-end][ set pcolor lime ]*

*;ask n-of 75 patches with [ distancexy 0 0 < ( world-width * 0.75 / 2 - 6) and distancexy 0 0 > ( world-width * 0.75 / 2 - 15 ) and pzcor >= mid-start and pzcor <= mid-end][ set pcolor lime ]*

*;ask n-of 55 patches with [ distancexy 0 0 < ( world-width * 0.75 / 2 - 6) and distancexy 0 0 > ( world-width * 0.75 / 2 - 15 ) and pzcor >= 4 and pzcor <= lower-end][ set pcolor lime ]*

*;ask n-of 20 patches with [ distancexy 0 0 < ( world-width * 0.75 / 2 - 15 ) and pzcor >= lower-start and pzcor <= 3 ][ set pcolor lime ]*

*]*

*; ***************Fruit in the edge part of Closed-canopy/Vase-shaped****************

*ask patches with [pxcor = 7 and pycor = -13 and pzcor = 5 ][set pcolor red]*

*ask patches with [pxcor = -10 and pycor = -3 and pzcor = 6 ][set pcolor red]*

*ask patches with [pxcor = 10 and pycor = 8 and pzcor = 15 ][set pcolor red]*

*ask patches with [pxcor = -16 and pycor = 5 and pzcor = 20 ][set pcolor red]*

*ask patches with [pxcor = -2 and pycor = -12 and pzcor = 22 ][set pcolor red]*

*ask patches with [pxcor = -5 and pycor = 15 and pzcor = 11 ][set pcolor red]*

*; *********************** Fruit in the central part of Closed-canopy ******************

*;ask patches with [pxcor = 2 and pycor = -4 and pzcor = 5 ][set pcolor red]*

*;ask patches with [pxcor = -3 and pycor = -1 and pzcor = 6 ][set pcolor red]*

*;ask patches with [pxcor = 3 and pycor = 2 and pzcor = 15 ][set pcolor red]*

*;ask patches with [pxcor = -2 and pycor = 3 and pzcor = 20 ][set pcolor red]*

*;ask patches with [pxcor = -2 and pycor = -1 and pzcor = 22 ][set pcolor red]*

*;ask patches with [pxcor = -2 and pycor = 2 and pzcor = 11 ][set pcolor red]*

*end*

*to go*

*if ticks >= 15 ;Simulation conducted within 15 mins*

*[*

*stop*

*] ;Simulation will stop after given ticks*

*set time ticks*

*if not any? turtles-on patches with [pcolor = lime or pcolor = red] ;Simulation stop if there are no any flies on the canopy*

*[*

*stop*

*]*

*move-flies ;Fruit flies start moving*

*tick*

*end*

*to move-flies ;Starting to move*

*ask flies [*

*set detection-radius 40 / 4 ;The length of detection-radius is 40 cm; Each cube represent 10 cm*

*;show detection-radius*

*set pitch 0 ;Set fruit flies position parallel with ground*

*set roll 0*

*if pcolor = red [ ;Found fruit, so stop*

*set count-down count-down - 1 ;Decrement timer*

*if count-down = 0*

*[*

*short-hop ;Short hops on canopy - Short hop procedure*

*reset-count-down*

*]*

*]*

*if pcolor = lime [ ;Find a leaf, so do short hops and always find the nearby leaves as the next destination*

*short-hop ; Short hops on canopy - Short hop procedure*

*]*

*]*

*end*

*to short-hop ; Short hop procedure*

*let reach-top count patches in-cone detection-radius 360 with [pcolor = lime or pcolor = red and self != [patch-here] of myself and pzcor > [ zcor ] of myself ] ;Creates 180 degree field of view for sensing the top part of canopy (based on a 360 degree sensing angle)*

*let patches-in-view count patches in-cone detection-radius 60 with [pcolor = lime or pcolor = red and self != [patch-here] of myself ] ;Sensing the edge part of canopy with a 60 degree sensing angle*

*ifelse reach-top != 0 [ ;There are leaves above fly*

*ifelse patches-in-view = 0 [ ;In the edge of canopy and only turn back towards certral parts of canopy, and then choose a target leaf*

*;write "***************************Edge**********************************"*

*let rand random 100*

*if rand < 20[ ; The 20% of flies will leave the canopy if they are in the edge of canopy*

*die*

*]*

*;Turning back towards central parts of canopy but not moving into it*

*let me self*

*let current-heading [ heading ] of me*

*;print "*************************current-heading***************************"*

*;show current-heading*

*set heading current-heading + 180*

*set heading vary (heading + 45) (heading - 45)*

*;print "***************************Turn back heading***********************"*

*;show heading*

*]*

*[ ;Not in the edge of canopy and should be able to sense around nearby leaves*

*set heading vary 0 360*

*]*

*;Sensing procedure*

*set pitch 90 ;Fruit flies face upwards*

*let closest-leaf one-of (patches in-cone detection-radius 220 with [pcolor = lime and self != [patch-here] of myself]) ;Set 220 degree - Sensing one of the around nearby leaves within the detection radius*

*let fruit one-of (patches in-cone detection-radius 360 with [pcolor = red and self != [patch-here] of myself]) ;Sensing fruit within the detection radius*

*set fruit-chosen-flag 0 ;Set none visit signal on the fruit*

*set pitch 0 ;Fruit flies face parallel with ground*

*face closest-leaf ;Fruit flies face one of nearby leaves*

*let destination-leaf min-one-of (patches in-cone detection-radius 30 with [pcolor = lime and self != [patch-here] of myself])[distance myself] ;Finding the nearest leaf in the direction where they are facing; Using a 30 degree sensing angle to narrow down facing direction/leaves in front of fruit flies*

*if is-patch? fruit ;If fruit presents within the detection radius/sensing sphere (sensory range)*

*[*

*let fxcor [pxcor] of fruit*

*let fycor [pycor] of fruit*

*let fzcor [pzcor] of fruit*

*facexyz fxcor fycor fzcor ;Fruit flies face fruit*

*let on-the-way-to-fruit min-one-of (patches in-cone detection-radius 30 with [pcolor = lime or pcolor = red and self != [patch-here] of myself])[distance myself] ;Finding the nearest leaf in the front of fruit. It would be fruit if there are no any leaves. Using a 30 degree sensing angle to narrow down facing direction and more accurate*

*let sxcor [pxcor] of on-the-way-to-fruit*

*let sycor [pycor] of on-the-way-to-fruit*

*let szcor [pzcor] of on-the-way-to-fruit*

*let search-step-length distancexyz sxcor sycor szcor ;Step length*

*let dist distancexyz fxcor fycor fzcor ;Distance to fruit*

*let pro random 100*

*let chance 163.65946 - 64.85154 * ln ( dist + 1.63641 ) ;The probability of locating fruit that is a function of distance*

*;print"**********************Distance****************************"*

*;show dist*

*;print"**********************Chance****************************"*

*;show chance*

*if pro < chance [*

*;print"**********************Distance****************************"*

*;show dist*

*;print"**********************Chance****************************"*

*;show chance*

*;show "25%"*

*;print"*******************Approaching to fruit*********************"*

*;show search-step-length*

*;print"*******************Approaching to fruit*********************"*

*;***********************Approaching to fruit***********************

*move-to on-the-way-to-fruit ;Move to the nearest leaf in the front of fruit or fruit*

*count-visits*

*set fruit-chosen-flag 1 ;Set visit signal on the fruit*

*]*

*]*

*;If fruit does not present within the detection radius and will move to the nearest leaf in the directon where they are facing.*

*if fruit-chosen-flag = 0 [*

*ifelse is-patch? destination-leaf ;If the nearest leaf presents within the detection radius*

*[ ;print "*******************Find the closest leaf***************************"*

*;Move to the nearest leaf*

*let nwxcor [pxcor] of destination-leaf*

*let nwycor [pycor] of destination-leaf*

*let nwzcor [pzcor] of destination-leaf*

*facexyz nwxcor nwycor nwzcor ;Faceing the nearest leaf*

*;print"************************Step Length****************************"*

*;show distancexyz nwxcor nwycor nwzcor ;step length*

*;print"************************Step Length****************************"*

*move-to destination-leaf ;Move to the nearest leaf in the directon where they are facing.*

*count-visits*

*]*

*[ ;If there is no the nearest leaf in the directon where they are facing, then randomly hop to find the nearest leaf or fruit*

*;print "******************************Hop*******************************"*

*random-jump ;Random hop procedure- maximum distance is detection-radius*

*let nearest-leaf min-one-of (patches with [pcolor = 65 or pcolor = red] ) [distance myself] ; After hopping, find the nearest leaf or fruit*

*if is-patch? nearest-leaf [*

*let nxcor [pxcor] of nearest-leaf*

*let nycor [pycor] of nearest-leaf*

*let nzcor [pzcor] of nearest-leaf*

*facexyz nxcor nycor nzcor*

*move-to nearest-leaf ;Move to the nearest leaf or fruit*

*;print "*************************nearest-leaf***************************"*

*count-visits*

*]*

*]*

*]*

*]*

*[ ;Else reached the top canopy and leave the tree*

*;print "*****************************Stop*********************************"*

*die*

*]*

*;reset-count-down ; Reset countdown ticks and ready to move*

*set step-length (step-length + 1)*

*;set step step-length*

*;show step*

*end*

*to-report vary [low high] ;Randomly generate value*

*report low + random (high - low + 1)*

*end*

*to reset-count-down*

*set count-down 1 ;Reset countdown ticks to 1 min, because flies will stay 1 min on leaf or fruit*

*end*

*to random-jump*

*;set heading vary 0 360 ; Heading direction randomly between 0 and 360 degree*

*set pitch vary 0 90 ; Pitch angle randomly - upwards according observed behavioural rules*

*jump vary 1 detection-radius*

*count-visits ; Count visits*

*end*

*to count-visits ;Show visits on leaves or fruit*

*if count turtles-here > 0 [*

*set counter (counter + 1)*

*]*

*if pcolor = lime or pcolor = red[*

*set plabel counter*

*]*

*end*

*to display-output ;Show gradient coloured outcomes*

*ask patches [*

*if counter > 0 and pcolor = lime [*

*set pcolor scale-color 125 counter 20 -2 ;1000 -200 600 -30*

*]*

*if counter > 0 and pcolor = red [*

*set pcolor scale-color 125 counter 20 -2 ;1000 -200 600 -30*

*]*

*]*

*end*

**Using Netlogo interface buttons is to display or clear hop paths of fruit flies.**

*;display*

*pen-down*

*;clear*

*clear-drawing*

**The use of Netlogo interface monitors is to display visits on foliage/fruit in each region (upper vs. middle vs. lower and inner vs. outer) and the number of fruit flies still on the tree.**

*;Visits on foliage in each region*

*;Upper*

*sum [plabel] of patches with [ pcolor = lime and pzcor >= upper-start and pzcor <= upper-end]*

*;Middle*

*sum [plabel] of patches with [ pcolor = lime and pzcor >= mid-start and pzcor <= mid-end]*

*;Lower*

*sum [plabel] of patches with [ pcolor = lime and pzcor >= lower-start and pzcor <= lower-end]*

*;Inner*

*sum [plabel] of patches with [ pcolor = lime and distancexy 0 0 < ( world-width * 0.75 / 2 - 12 ) and pzcor >= lower-start and pzcor <= upper-end]*

*;Outer*

*sum [plabel] of patches with [ pcolor = lime and distancexy 0 0 > ( world-width * 0.75 / 2 - 12 ) and distancexy 0 0 < ( world-width * 0.75 / 2 - 6 ) and pzcor >= lower-start and pzcor <= upper-end]*

*; Visits on fruit in each region*

*;Upper*

*sum [plabel] of patches with [ pcolor = red and pzcor >= upper-start and pzcor <= upper-end]*

*;Middle*

*sum [plabel] of patches with [ pcolor = red and pzcor >= mid-start and pzcor <= mid-end]*

*;Lower*

*sum [plabel] of patches with [ pcolor = red and pzcor >= lower-start and pzcor <= lower-end]*

*;The number of fruit flies still on the tree*

*count turtles*
