# Supplementary material for: A Generic Individual-Based Spatially Explicit Model as a Novel Tool for Investigating Insect-Plant Interactions: A Case Study of the Behavioural Ecology of Frugivorous Tephritidae
Source: PLoS One. 2016 Mar 21;11(3):e0151777. doi: 10.1371/journal.pone.0151777 (PMC4801379; doi:10.1371/journal.pone.0151777)
Supplement: S2 Appendix — (DOCX) [file pone.0151777.s002.docx]

**S2 Appendix: Model Analysis**

**Sensitivity analysis**

The detection radius parameter for the Queensland fruit fly (Qfly), *Bactrocera tryoni*, has not been studied in the literature, therefore a sensitivity analysis was performed to fine tune its value in the model. We use 80 cm as the initial value for this parameter, as the distance threshold of the sphere of attraction for a related species, the apple maggot fly, *Rhagoletis pomonella* (Walsh), is around 80 cm in an apple tree canopy [1].

The model used one fly for each simulation in order to be consistent with the experiments [2], and was run 100 times. When the initial value (80 cm) was used in the model, a pattern was produced with too many flies remaining in the tree, which did not match with published experimental outcomes. We followed the POM strategy [3] and protocol [4], and used observed patterns [2] at multiple scales to reduce the uncertainty in this parameter [5]. Patterns are listed as follow:

- Pattern1: most visits occur in the inner part of the tree.
- Pattern2: most flies (over 80%) leave the tree within 15 minutes.
- Pattern3: the mean number of visits per fly on tree foliage is 8.9 (SD = 4.802) found in experiments.

Therefore, we tried a range of values (20 cm, 40 cm and 60 cm) for this parameter to reproduce the observed patterns (Tables 1-4 and Fig. 1).

**Table 1. The outcomes for Pattern1 using different detection radius values**

| **Pattern1** | **Inner** | **Outer** | **Inner** | **Outer** |  |  |
| --- | --- | --- | --- | --- | --- | --- |
|  | **Mean** | | **SD** | | **t** | **P** |
| 20 cm | 2.33 | 1.59 | 3.49 | 2.567 | 1.76 | 0.082 |
| 40 cm | 5.12 | 3.72 | 3.627 | 2.519 | 3.04 | **0.003** |
| 60 cm | 6.43 | 3.21 | 4.164 | 2.28 | 7.45 | **< 0.001** |
| 80 cm | 7.86 | 2.27 | 4.233 | 1.841 | 12.37 | **< 0.001** |

**Table 2. The outcomes for Pattern2 using different detection radius values**

| **Pattern2** | **Percentage of flies left the tree within 15 mins** |
| --- | --- |
| 20 cm | **95%** |
| 40 cm | **81%** |
| 60 cm | 67% |
| 80 cm | 65% |

**Table 3. The outcomes for Pattern3 using different detection radius values**

| **Pattern3** | Field studies | 20 cm | Field studies | 40 cm | Field studies | 60 cm | Field studies | 80 cm |
| --- | --- | --- | --- | --- | --- | --- | --- | --- |
| **Mean** | 8.9 | 3.92 | 8.9 | 8.84 | 8.9 | 9.64 | 8.9 | 10.13 |
| **SD** | 4.802 | 4.446 | 4.802 | 4.223 | 4.802 | 5.138 | 4.802 | 4.713 |
| **t** | 4.86 | | **0.06** | | **-0.70** | | **-1.19** | |
| **df** | 38 | | **37** | | **43** | | **40** | |
| **P** | < 0.001 | | **0.953** | | **0.488** | | **0.243** | |

**Table 4. The outcomes of all patterns using different detection radius values**

| **Parameter Values** | **Pattern1** | **Pattern2** | **Pattern3** |
| --- | --- | --- | --- |
| 20 cm | t = 1.76 P = 0.082 | **95%** | t = 4.86 df = 38 P < 0.001 |
| 40 cm | **t = 3.04 P = 0.003** | **81%** | **t = 0.06 df = 37 P = 0.953** |
| 60 cm | **t = 7.45 P < 0.001** | 67% | **t = -0.70 df = 43 P = 0.488** |
| 80 cm | **t = 12.37 P < 0.001** | 65% | **t = -1.19 df = 40 P = 0.243** |

b

a

b

a

**Fig. 1.** **Mean time (min) spent on host fruit by a fly in the closed-canopy using different detection radius values.** The one-way ANOVA followed by the Tukey test: 20 cm vs. 40 cm vs. 60 cm vs. 80 cm (Mean = 0.28|1.21|0.92|0.49, F = 16.99, df = 3, P < 0.001). Columns surmounted with the same letter are not significantly different at P = 0.05 (n = 100 simulations).

As we can see from above tables, simulations with 40 cm led to the best correspondence to what has been observed in reality, reproducing all observed patterns from the experiments [2]. Our model also suggested that detection radius (40 cm) for fruit flies is the optimal distance in this tree canopy, allowing them to find more fruit in the tree (Fig. 1). Thus, we used 40 cm for detection radius of the Qfly in the following predictive simulation experiments.

**Robustness Analysis**

We performed a robustness analysis to identify conditions under which the modified model no longer reproduces one or more of the patterns, to help us to improve our understanding of the control mechanisms in the model, as suggested by Grimm and Berger [6]. We created different versions of our model by changing its structure, and used two of the heuristics for analyzing IBMs suggested by Railsback and Grimm [7]: analyzing simplified versions of the model and exploring unrealistic scenarios. Versions of our model are as follows:

- Version1: we changed the sensing angle (field of view) for sensing foliage from 220° to 360°.
- Version2: we removed host fruit from the canopy.

The model used 30 flies run for 15 time steps (equivalent to 15 minutes in reality) for each simulation. This was replicated 50 times for each version; the outcomes are shown in Fig. 2 and Fig. 3. We also compared these two versions to our original model to identify which control mechanisms cause our model to reproduce one or more of observed patterns (Table 5 and Table 6).

c

a

b

**Fig. 2.** **Mean time (min) spent by 30 *Bactrocera tryoni* on the vegetation volume in the closed-canopy in the model version1.** The one-way ANOVA followed by the Tukey test: upper vs. middle vs. lower (Mean = 34.42|108.98|147.14, F = 479.61, df = 2, P < 0.001). Columns surmounted with the same letter are not significantly different at P = 0.05 (n = 50 simulations).

c

b

a

**Fig. 3.** **Mean time (min) spent by 30 *Bactrocera tryoni* on the vegetation volume in the closed-canopy in the model version2.** The one-way ANOVA followed by the Tukey test: upper vs. middle vs. lower (Mean = 111.96|95.94|54.90, F = 166.48, df = 2, P < 0.001). Columns surmounted with the same letter are not significantly different at P = 0.05 (n = 50 simulations).

**Table 5. The comparison of mean time (min) spent by 30 *Bactrocera tryoni* on the vegetation volume and host fruit in each part of closed-canopy between original version and version1.**

| **Closed-canopy** | **Original Version** | **Version1** |  |  |  |
| --- | --- | --- | --- | --- | --- |
|  | **Mean** | | **t** | **df** | **P** |
| Upper | 103.7 | 34.42 | 20.90 | 76 | **< 0.001** |
| Middle | 97.24 | 108.98 | -3.71 | 94 | **< 0.001** |
| Lower | 55.58 | 147.14 | -24.17 | 67 | **< 0.001** |
| Fruit | 37.98 | 40.02 | -1.26 | 97 | 0.211 |

**Table 6. The comparison of mean time (min) spent by 30 *Bactrocera tryoni* on the vegetation volume in each part of closed-canopy between original version and version2.**

| **Closed-canopy** | **Original Version** | **Version2** |  |  |  |
| --- | --- | --- | --- | --- | --- |
|  | **Mean** | | **t** | **df** | **P** |
| Upper | 103.7 | 111.96 | -1.97 | 97 | 0.052 |
| Middle | 97.24 | 95.94 | 0.45 | 97 | 0.656 |
| Lower | 55.58 | 54.9 | 0.33 | 96 | 0.744 |

After the robustness analysis, only version1 breaks observed patterns. Thus, totally random choice of direction, instead of a mainly upward choice is an unrealistic scenario. In contrast to the original model, simplified version (version2) does not show any differences, and it does not break observed patterns. This would suggest that host fruit has less contribution to fruit fly movement patterns than plant architecture.

In summary, the sensitivity and robustness analysis show that upward movements combined with detection radius (optimal short hop distance) have a strong influence on fruit fly movement and spatial distribution patterns, in agreement with our hypotheses that spatial patterns of insect abundance in plant canopies emerge from the behaviour of individual insects.

**References:**

1. Verdeny-Vilalta O, Aluja M, Casas J. Relative roles of resource stimulus and vegetation architecture on the paths of flies foraging for fruit. Oikos. 2015;124(3):337-46.

2. Dalby-Ball G, Meats A. Effects of fruit abundance within a tree canopy on the behaviour of wild and cultured Queensland fruit flies, *Bactrocera tryoni* (Froggatt) (Diptera: Tephritidae). Australian Journal of Entomology. 2000;39(3):201-7.

3. Grimm V, Railsback SF. Pattern-oriented modelling: a ‘multi-scope’ for predictive systems ecology. Philosophical Transactions of the Royal Society B: Biological Sciences. 2012;367(1586):298-310.

4. Wiegand T, Jeltsch F, Hanski I, Grimm V. Using pattern‐oriented modeling for revealing hidden information: a key for reconciling ecological theory and application. Oikos. 2003;100(2):209-22.

5. Grimm V, Revilla E, Berger U, Jeltsch F, Mooij WM, Railsback SF, et al. Pattern-oriented modeling of agent-based complex systems: lessons from ecology. Science. 2005;310(5750):987-91.

6. Grimm V, Berger U. Robustness analysis: Deconstructing computational models for ecological theory and applications. Ecological Modelling. 2015.

7. Railsback SF, Grimm V. Agent-based and individual-based modeling: a practical introduction: Princeton University Press; 2011.
