# Supplementary material for: A Generic Individual-Based Spatially Explicit Model as a Novel Tool for Investigating Insect-Plant Interactions: A Case Study of the Behavioural Ecology of Frugivorous Tephritidae
Source: PLoS One. 2016 Mar 21;11(3):e0151777. doi: 10.1371/journal.pone.0151777 (PMC4801379; doi:10.1371/journal.pone.0151777)
Supplement: S3 Appendix — (DOCX) [file pone.0151777.s003.docx]

**S3 Appendix: Field Study**

**Introduction**

Our field study was conducted to test whether model predictions were realised when using real insects in a controlled field environment. The purpose was to determine whether our generic individual-based spatially explicit model demonstrated reliable outputs in the real world and is therefore trust-worthy. Pattern-oriented models should be able to produce comparative predictions that can be tested in the field [1, 2].

**Methods**

*Insect Rearing and Holding*

The Queensland fruit fly, *Bactrocera tryoni*, used in experimentation, were obtained from a population maintained at Queensland University of Technology - fruit flies were originally sourced from the State Department of Agriculture and Fisheries, Brisbane, Queensland, Australia. The fruit flies were housed in mesh cages (35 cm x 25 cm x 30 cm) containing some artificial tree branches in the insectary at the University of Queensland. The insectary was under 12h light (7am to 7pm) and 12h dark artificial light conditions, and exposed to natural light during daylight. The fruit flies were maintained at 27°C and provided with sugar, hydrolysed yeast (to enable egg development), and water *ad libitum*.

*Experiments*

All experiments used mated, oviposition-ready females (14 - 25 days old) [3]. Trials ran between 11:00am and 3:30pm, from 25^th^ September to 02^nd^ October 2015, on sunny days. Temperatures ranged between 15.8°C and 29.1°C during all experiments (temperature data recorded every minute; The School of Geography, Planning and Environmental Management, The University of Queensland).

Experiments were conducted in a custom-built, 2.5 m x 2.5 m x 2 m mesh-covered walk-in cage (Fig. 1), located in the field at the University of Queensland, St Lucia campus (Latitude: 27.496570° S, Longitude: 153.018222° E). The walk-in cage contained two artificial trees with different canopy architectures, modified from artificial apple trees (Sharyn’s Greenery Design, Underwood, Australia). One had a closed-canopy of approximately 1.05 m diameter by 1 m height; the other was a vase-shaped tree with its canopy approximately 1.2 m in width and 1 m in height (Fig. 2). Artificial trees have been successfully used to study fruit fly behaviour before [4, 5].


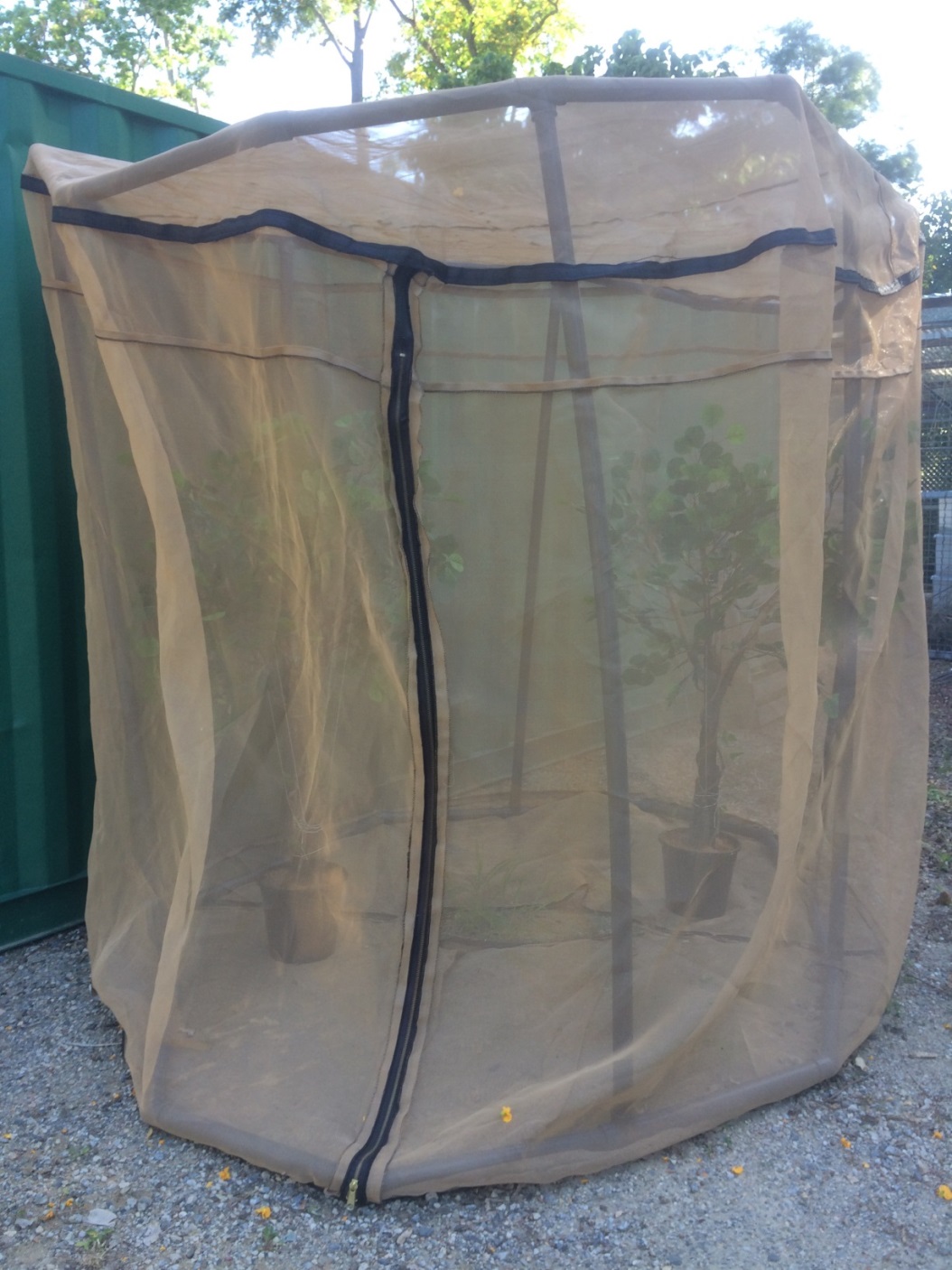


**Fig. 1.** **The 2.5 m x 2.5 m x 2 m mesh-covered walk-in cage.** It contained two artificial trees (left: the closed-canopy vs. right: the vase-shaped; see Fig. 2) and was used to test behaviour of mated, oviposition-ready, female *Bactrocera tryoni* for fruit preference between the two different plant architectures.


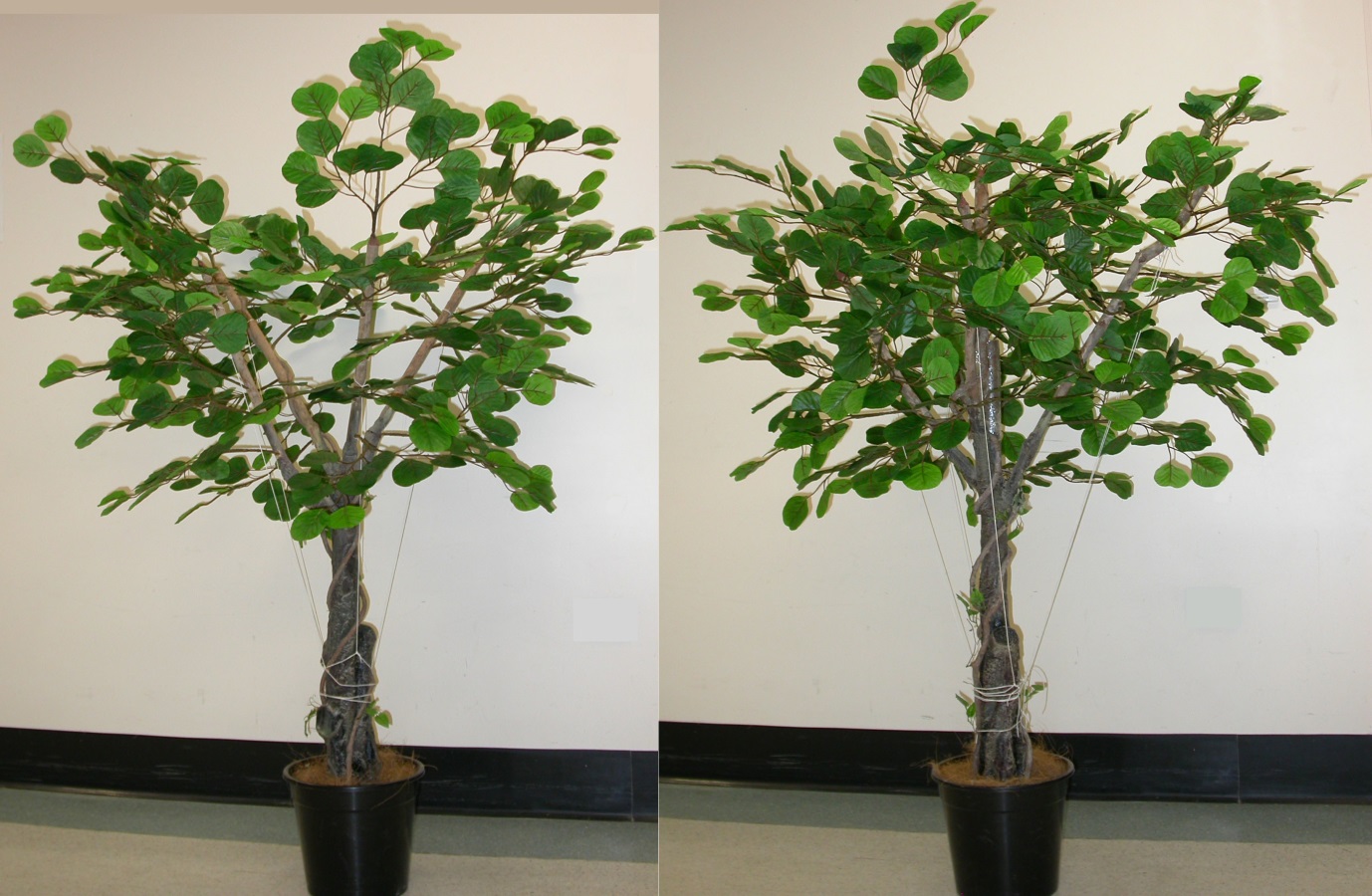


**Fig. 2.** **Two trees with different plant architectures.** The left one has been shaped to be a vase-shaped tree with a canopy approximately 1.2 m in width and 1 m in height, and the right one has a closed-canopy of approximately 1.05 m diameter by 1 m height.

In the morning of each experimental day, female fruit flies were collected and moved into smaller mesh-covered cages (20 cm x 15 cm x 15 cm) ready for transportation, and each cage contained 20 mated females. For each release, the small cage was placed on the ground in the walk-in cage, where flies had the same distance to both artificial trees. The small cage was then opened and the 20 female fruit flies were released and free to move around the walk-in cage at their own will. After 10 minutes, a grape-shaped tomato, approximately 2 cm diameter by 4 cm length, *Solanum lycopersicum* variety Roma, was placed in the upper, middle and lower regions in each tree (three grape tomatoes in total on each tree). This time delay allowed the fruit flies to have time to acclimate to the walk-in cage environment. The positioning of two trees maximised the observer’s ability to see all grape tomatoes from the observer standing point, which reducing movement and therefore possible disturbance of fruit flies.

Once a fly landed on a fruit, the time was noted as was the plant architecture choice. The fruit fly was then immediately, gently removed using forceps and put into a holding cage, to ensure its preference of plant architectures was only recorded once. Each set of flies was allowed 20 minutes to choose a fruit. Not all flies made a landing. After this time, all fruit flies were removed from the walk-in cage and a new set of flies released.

*Data Analysis*

Data collected from the experiment was analysed using a Chi-square goodness-of-fit test for observed visits on host fruit to assess proportions between trees. This allowed us to determine whether any plant architecture choice was favoured over another.

**Results**

There were significantly more visits on host fruit in the vase-shaped tree than on host fruit in the closed-canopy tree (X^2^ = 10.051, df = 1, P = 0.002) (Fig. 3). Thus, the vase-shaped tree was preferred over the closed-canopy tree. The result is in accordance with prediction from the generic individual-based spatially explicit model, indicating that model outputs are trust-worthy.

**Fig. 3.** **The results of field study.** The total number of visits on host fruit in each tree. Chi-square goodness-of-fit test for observed visits on host fruit was used to look at the differences between trees architectures. There were significantly more visits on host fruit in the vase-shaped tree than on host fruit in the closed-canopy tree (X^2^ = 10.051, df = 1, P = 0.002). ** Significance at P = 0.002.

**References**

1. Grimm V, Railsback SF. Pattern-oriented modelling: a ‘multi-scope’ for predictive systems ecology. Philosophical Transactions of the Royal Society B: Biological Sciences. 2012;367(1586):298-310.

2. Wiegand T, Jeltsch F, Hanski I, Grimm V. Using pattern‐oriented modeling for revealing hidden information: a key for reconciling ecological theory and application. Oikos. 2003;100(2):209-22.

3. Dalby-Ball G, Meats A. Effects of fruit abundance within a tree canopy on the behaviour of wild and cultured Queensland fruit flies, *Bactrocera tryoni* (Froggatt) (Diptera: Tephritidae). Australian Journal of Entomology. 2000;39(3):201-7.

4. Raghu S, Clarke AR. Spatial and temporal partitioning of behaviour by adult dacines: direct evidence for methyl eugenol as a mate rendezvous cue for *Bactrocera cacuminata*. Physiological Entomology. 2003;28(3):175-84.

5. Balagawi S, Jackson K, Clarke A. Resting sites, edge effects and dispersion of a polyphagous *Bactrocera* fruit fly within crops of different architecture. Journal of Applied Entomology. 2014;138(7):510-8.
